# MAGMA: Generalized Gene-Set Analysis of GWAS Data

**DOI:** 10.1371/journal.pcbi.1004219

**Published:** 2015-04-17

**Authors:** Christiaan A. de Leeuw, Joris M. Mooij, Tom Heskes, Danielle Posthuma

**Affiliations:** 1 Department of Complex Trait Genetics, Center for Neurogenomics and Cognitive Research, VU University Amsterdam, Amsterdam, The Netherlands; 2 Institute for Computing and Information Sciences, Radboud University Nijmegen, Nijmegen, The Netherlands; 3 Informatics Institute, University of Amsterdam, Amsterdam, The Netherlands; 4 Department of Clinical Genetics, VU University Medical Centre Amsterdam, Neuroscience Campus Amsterdam, The Netherlands; Stanford University, UNITED STATES

## Abstract

By aggregating data for complex traits in a biologically meaningful way, gene and gene-set analysis constitute a valuable addition to single-marker analysis. However, although various methods for gene and gene-set analysis currently exist, they generally suffer from a number of issues. Statistical power for most methods is strongly affected by linkage disequilibrium between markers, multi-marker associations are often hard to detect, and the reliance on permutation to compute p-values tends to make the analysis computationally very expensive. To address these issues we have developed MAGMA, a novel tool for gene and gene-set analysis. The gene analysis is based on a multiple regression model, to provide better statistical performance. The gene-set analysis is built as a separate layer around the gene analysis for additional flexibility. This gene-set analysis also uses a regression structure to allow generalization to analysis of continuous properties of genes and simultaneous analysis of multiple gene sets and other gene properties. Simulations and an analysis of Crohn’s Disease data are used to evaluate the performance of MAGMA and to compare it to a number of other gene and gene-set analysis tools. The results show that MAGMA has significantly more power than other tools for both the gene and the gene-set analysis, identifying more genes and gene sets associated with Crohn’s Disease while maintaining a correct type 1 error rate. Moreover, the MAGMA analysis of the Crohn’s Disease data was found to be considerably faster as well.

## Author Summary

Gene and gene-set analysis are statistical methods for analysing multiple genetic markers simultaneously to determine their joint effect. These methods can be used when the effects of individual markers is too weak to detect, which is a common problem when studying polygenic traits. Moreover, gene-set analysis can provide additional insight into functional and biological mechanisms underlying the genetic component of a trait. Although a number of methods for gene and gene-set analysis are available however, they generally suffer from various statistical issues and can be very time-consuming to run. We have therefore developed a new method called MAGMA to address these issues, and have compared it to a number of existing tools. Our results show that MAGMA detects more associated genes and gene-sets than other methods, and is also considerably faster. The way the method is set up also makes it highly flexible. This makes it suitable as a basis for more general statistical analyses aimed at investigating more complex research questions.

## Introduction

In the past decade, genome-wide association studies (GWAS) have successfully identified new genetic variants for a wide variety of phenotypes [1]. However, despite growing sample sizes, the genetic variants discovered by GWAS generally account for only a fraction of the total heritability of a phenotype [2,3]. More than anything, GWAS has shown that many phenotypes, such as height [4], schizophrenia [5] and BMI [6] are highly polygenic and influenced by thousands of genetic variants with small individual effects, requiring very large sample sizes to detect them.

Gene and gene-set analysis have been suggested as potentially more powerful alternatives to the typical single-SNP analyses performed in GWAS [7]. In gene analysis, genetic marker data is aggregated to the level of whole genes, testing the joint association of all markers in the gene with the phenotype. Similarly, in gene-set analysis individual genes are aggregated to groups of genes sharing certain biological, functional or other characteristics. Such aggregation has the advantage of considerably reducing the number of tests that need to be performed, and makes it possible to detect effects consisting of multiple weaker associations that would otherwise be missed. Moreover, gene-set analysis can provide insight into the involvement of specific biological pathways or cellular functions in the genetic etiology of a phenotype. Gene-set analysis methods can be subdivided into self-contained and competitive analysis, with the self-contained type testing whether the gene set contains any association at all, and the competitive type testing whether the association in the gene set is greater than in other genes [7].

Various methods for gene and gene-set analysis are currently available [7–13]. However, one concern with most existing methods is that they first summarize associations per marker before aggregating them to genes or gene sets. As demonstrated by Moskvina et al. this makes the statistical power strongly dependent on local linkage disequilibrium (LD) [14], and also reduces power to detect associations dependent on multiple markers.

An additional concern is that current gene-set analysis methods generally use a permutation-based approach. These are often very computationally demanding, and since no parametric model is used it is often not made explicit which null hypothesis is being evaluated and what assumptions are made. This makes it more difficult to determine the properties of the analysis such as how the significance of a gene set relates to the significance of its constituent genes or whether the analysis corrects for a polygenic architecture. This complicates the interpretation of results and hampers comparison between results of different gene-set analysis methods.

To address such issues we have developed MAGMA (Multi-marker Analysis of GenoMic Annotation), a fast and flexible tool for gene and gene-set analysis of GWAS genotype data. MAGMA’s gene analysis uses a multiple regression approach to properly incorporate LD between markers and to detect multi-marker effects. The gene-set analysis is built as a distinct layer around this gene analysis, providing the flexibility to independently change and expand both the gene and the gene-set analysis. Both self-contained and competitive gene-set analyses are implemented using a gene-level regression model. This regression approach offers a generalized framework which can also analyse continuous gene properties such as gene expression levels as well as conditional analyses of gene sets and other gene properties, and which can be extended to allow joint and interaction analysis of multiple gene sets and other gene properties as well. More traditional gene analysis models are also implemented, for comparison and to provide analysis of SNP summary statistics.

To evaluate the performance of MAGMA we have applied it to the Wellcome Trust Case-Control Consortium (WTCCC) Crohn’s Disease (CD) GWAS data-set [15], using the MSigDB Canonical Pathways [16] for the gene-set analysis. Simulation studies were performed to verify type 1 error rates for MAGMA. The CD data set was then analysed using MAGMA and with five commonly used other tools for gene and gene-set analyses, specifically VEGAS [17], PLINK [8], ALIGATOR [9], INRICH [10] and MAGENTA [12]. The results show that MAGMA has greater statistical power than the other methods, while also being considerably faster.

## Materials and Methods

### Model structure

The gene-set analysis is divided into two distinct and largely independent parts. In the first part a gene analysis is performed to quantify the degree of association each gene has with the phenotype. In addition the correlations between genes are estimated. These correlations reflect the LD between genes, and are needed in order to compensate for the dependencies between genes during the gene-set analysis. The gene p-values and gene correlation matrix are then used in the second part to perform the actual gene-set analysis.

The advantage of decoupling these two parts of the analysis in this manner is that each can be changed independently from the other, simplifying the development of changes and extensions to either part of the model. Moreover, since the second part only uses the output from the first part and not the raw genotype data they do not need to be performed at the same time or place, making it much more straightforward to perform multiple gene-set analyses on the same data or to analyse multiple data sets across a large-scale collaboration.

### Gene analysis

The gene analysis in MAGMA is based on a multiple linear principal components regression [18] model, using an F-test to compute the gene p-value. This model first projects the SNP matrix for a gene onto its principal components (PC), pruning away PCs with very small eigenvalues, and then uses those PCs as predictors for the phenotype in the linear regression model. This improves power by removing redundant parameters, and guarantees that the model is identifiable in the presence of highly collinear SNPs. By default only 0.1% of the variance in the SNP data matrix is pruned away.

With $X_{g}^{*}$ the matrix of PCs, *Y* the phenotype and *W* an optional matrix of covariates the model can thus be written as $Y=\alpha_{0g}\vec{1}+X_{g}^{*}\alpha_{g}+W\beta_{g}+\varepsilon_{g}$, where the parameter vector *α*_*g*_ represents the genetic effect, *β*_*g*_ the effect of the optional covariates, *α*_0*g*_ the intercept and *ε*_*g*_ the vector of residuals. The F-test uses the null-hypothesis *H*_0_: $\alpha_{g}=\vec{0}$of no effect of gene *g* on the phenotype *Y*, conditional on all covariates.

This choice of gene analysis model is motivated by a balance of statistical and practical concerns. This multiple regression model ensures that LD between SNPs is fully accounted for. It also offers the flexibility to accommodate additional covariates and interaction terms as needed without changing the model. At the same time, since the F-test has a known asymptotic sampling distribution the gene p-values take very little time to compute, making the gene analysis much faster than permutation-based alternatives.

The linear regression model is also applied when *Y* is a binary phenotype. Although this violates some assumptions of the F-test, comparison of the F-test p-values with p-values based on permutation of the F-statistic shows that the F-test remains accurate (see ‘Supplemental Methods—Implementation Details’). MAGMA therefore uses the asymptotic F-test p-values by default, though it also offers an option to compute permutation-based p-values using an adaptive permutation procedure. In addition, comparison with logistic regression models shows that the results of the linear model are effectively equivalent to that of the more conventional logistic regression model, but without the computational cost.

### Gene-set analysis

To perform the gene-set analysis, for each gene *g* the gene p-value *p*_*g*_ computed with the gene analysis is converted to a Z-value *z*_*g*_ = Φ^−1^(1 – *p*_*g*_), where Φ^−1^ is the probit function. This yields a roughly normally distributed variable *Z* with elements *z*_*g*_ that reflects the strength of the association each gene has with the phenotype, with higher values corresponding to stronger associations.

Self-contained gene-set analysis tests whether the genes in a gene-set are jointly associated with the phenotype of interest. As such, using this variable *Z* a very simple intercept-only linear regression model can now be formulated for each gene set *s* of the form$Z_{s}=\beta_{0}\vec{1}+\varepsilon_{s}$, where *Z*_*s*_ is the subvector of *Z* corresponding to the genes in *s*. Evaluating *β*_0_ = 0 against the alternative *β*_0_ > 0 yields a self-contained test, since under the self-contained null hypothesis that none of the genes is associated with the phenotype *z*_*g*_ has a standard normal distribution for every gene *g*.

Competitive gene-set analysis tests whether the genes in a gene-set are more strongly associated with the phenotype of interest than other genes. To test this within the regression framework the model is first expanded to include all genes in the data. A binary indicator variable *S*_*s*_ with elements *s*_*g*_ is then defined, with *s*_*g*_ = 1 for each gene *g* in gene set *s* and 0 otherwise. Adding *S*_*s*_ as a predictor of *Z* yields the model$Z=\beta_{0s}\vec{1}+S_{s}\beta_{s}+\varepsilon$. The parameter *β*_*s*_ in this model reflects the difference in association between genes in the gene set and genes outside the gene set, and consequently testing the null hypothesis *β*_*s*_ = 0 against the one-sided alternative *β*_*s*_ > 0 provides a competitive test. Note that this is equivalent to a one-sided two-sample t-test comparing the mean association of gene-set genes with the mean association of genes not in the gene-set. Similarly, the self-contained analysis is equivalent to a one-sided single-sample t-test comparing the mean association of gene-set genes to 0.

It should be clear that in this framework, the gene-set analysis models are a specific instance of a more general gene-level regression model of the form$Z=\beta_{0}\vec{1}+C_{1}\beta_{1}+C_{2}\beta_{2}+\ldots +\varepsilon$. The variables *C*_*1*_, *C*_*2*_, …, in this generalized gene-set analysis model can reflect any gene property, from the binary indicators used for the competitive gene-set analysis to continuous variables such as gene size and expression levels. Any transformations of, and interactions between, such gene properties can also be added. This generalized gene-set analysis model thus allows for testing of conditional, joint and interaction effects of any combination of gene sets and other gene properties. In practice, the competitive gene-set analysis implemented in MAGMA in fact uses such a generalized model by default, performing a conditional test of *β*_*s*_ corrected for the potentially confounding effects of gene size, gene density and (if applicable, e.g. in meta-analysis) difference in underlying sample size, if such effects are present. This is achieved by adding these variables, as well as the log of these variables, as covariates to the gene-level regression model. The gene density is defined as the ratio of effective gene size to the total number of SNPs in the gene, with the effective gene size in turn defined as the number of principal components that remain after pruning.

One complication that arises in this gene-level regression framework is that the standard linear regression model assumes that the error terms have independent normal distributions, i.e.$\varepsilon \sim \text{MVN}(\vec{0},\sigma^{2}I)$. However, due to LD, neighbouring genes will generally be correlated, violating this assumption. This issue can be addressed by using Generalized Least Squares approach instead, and assuming that $\varepsilon \sim \text{MVN}(\vec{0},\sigma^{2}R)$. In MAGMA, the required gene-gene correlation matrix *R* is approximated by using the correlations between the model sum of squares (SSM) of each pair of genes from the gene analysis multiple regression model, under their joint null hypothesis of no association. These correlations are a function of the correlations between the SNPs in each pair of genes and thus provide a good reflection of the LD, and since they have a convenient closed-form solution they are easy to compute (see also ‘Supplemental Methods—Implementation Details’). Note that for the self-contained analysis, the submatrix *R*_*s*_ corresponding to only the genes in the gene set is used instead of *R*. In addition, since the self-contained null hypothesis guarantees that all *z*_*g*_ have a standard normal distribution, the error variance *σ* ^2^ can be set to 1.

### Analysis of summary SNP statistics

Since raw genotype data may not always be available for analysis, MAGMA also provides more traditional SNP-wise gene analysis models of the type implemented in PLINK and VEGAS. These SNP-wise models first analyse the individual SNPs in a gene and combine the resulting SNP p-values into a gene test-statistic, and can thus be used even when only the SNP p-values are available. Although evaluation of the gene test-statistic does require an estimate of the LD between SNPs in the gene, estimates based on reference data with similar ancestry as the data the SNP p-values were computed from has been shown to yield accurate results [17,19].

Two types of gene test statistics have been implemented in MAGMA: the mean of the *χ* ^2^ statistic for the SNPs in a gene, and the top *χ* ^2^ statistic among the SNPs in a gene. For the mean *χ* ^2^ statistic, a gene p-value is then obtained by using a known approximation of the sampling distribution [20,21]. For the top *χ* ^2^ statistic such an approximation is not available, and therefore an adaptive permutation procedure is used to obtain an empirical gene p-value. A random phenotype is first generated for the reference data, drawing from the standard normal distribution. This is then permuted, and for each permutation the top *χ* ^2^ statistic is computed for every gene. The empirical p-value for a gene is then computed as the proportion of permuted top *χ* ^2^ statistics for that gene that are higher than its observed top *χ* ^2^ statistic. The required number of permutations is determined adaptively for each gene during the analysis, to increase computational efficiency. Further details can be found in ‘Supplemental Methods—SNP-wise gene analysis’.

The MAGMA SNP-wise models can also be used to analyse raw genotype data, in which case the raw genotype data takes the place of the reference data and the SNP p-values are computed internally. Gene-set analysis based on these SNP-wise models proceeds in the same way as the gene-set analysis based on the multiple regression gene analysis model. The gene p-values resulting from the analysis are converted to Z-values in the same way to serve as input for the gene-set analysis. Similarly, the gene-gene correlation matrix *R* is obtained using the same formula as with the multiple regression model, but using the reference data to compute it.

### Other features and implementation

A number of additional features has been implemented in MAGMA, more fully described in ‘Supplemental Methods—Extensions’. Gene analysis can be expanded with a gene-environment interaction component, which can subsequently be carried over to the gene-set analysis. Options for aggregation of rare variants and for fixed-effects meta-analysis for both gene and gene-set analysis are also available. Efficient SNP to gene annotation and a batch mode for parallel processing are provided to simplify the overall analysis process. MAGMA is distributed as a standalone application using a command-line interface. The C++ source code is also made available, under an open source license. MAGMA can be downloaded from http://ctglab.nl/software/magma.

### Data

To evaluate the performance of MAGMA, the WTCCC Crohn’s Disease (CD) GWAS data [15] in conjunction with both WTCCC control samples was used. The data was cleaned according to the protocol described by Anderson [22], resulting in a sample of 1,694 cases and 2,917 controls with data for 403,227 SNPs. The European samples from the 1,000 Genomes data [23] and the HapMap 3 data [24] were used as reference data sets for the summary statistics gene analysis.

SNPs were annotated to genes based on dbSNP version 135 SNP locations and NCBI 37.3 gene definitions. For the main analyses only SNPs located between a gene’s transcription start and stop sites were annotated to that gene, yielding 13,172 protein-coding genes containing at least one SNP in the CD data. An additional annotation using a 10 kilobase window around each gene was made, yielding 16,970 genes, to determine the effect of using a window on relative performance. These two gene annotations were used for all analyses, to ensure that differences in default annotation settings did not cloud the comparison between tools. The 1,320 Canonical Pathways from the MSigDB database [16] were used for the gene-set analysis. The relatively large number of gene sets and the fact that the MSigDB Canonical Pathways are drawn from a number of different gene-set databases ensures a wide variety of gene sets, which should prevent the results from being too dependent on the choice of gene-set database.

### Analysis of CD data

The MAGMA gene analysis was performed on the raw CD data using the PC regression model (MAGMA-main). Gene analyses with VEGAS and PLINK were performed using the mean SNP statistic for VEGAS and both the mean SNP statistic (PLINK-avg) and the top SNP statistic (PLINK-top) for PLINK. Pruning in PLINK was turned off for these analyses. An additional PLINK analysis using the mean SNP statistic with pruning set to its default (PLINK-prune) was performed as well.

To facilitate the comparison, several additional SNP-wise gene-set analyses were performed in MAGMA with test-statistics matching those of PLINK-avg, PLINK-top and VEGAS: mean *χ* ^2^ (MAGMA-mean) and top *χ* ^2^ (MAGMA-top) on the raw CD data to match the two PLINK analyses, and mean *χ* ^2^ using CD SNP p-values and with either HapMap reference data (MAGMA-pval) to match VEGAS or with 1,000 Genomes reference data (MAGMA-pval-1K). The SNP summary statistics used for VEGAS and MAGMA-pval were computed using PLINK ‘--assoc’.

Gene-set analysis for MAGMA was performed based on the PC regression gene analysis model (MAGMA-main) as well as on the SNP-wise model with 1,000 Genomes reference data (MAGMA-pval-1K). Several other analyses were performed for comparison: PLINK self-contained gene-set analysis without pruning (PLINK-avg) and with pruning (PLINK-prune), as well as ALIGATOR, INRICH and MAGENTA competitive gene-set analysis. PLINK operates on raw genotype data, whereas all three competitive methods require only SNP p-values as input. No correction for stratification was used in any of the analyses except when explicitly specified. An overview of all analyses is given in Table 1.

**Table 1:** Overview of Crohn’s Disease analyses.

Name	Analysis	Input	Settings
MAGMA-main	gene, self-cont., comp.	Raw data	Multiple regression model (per gene)
MAGMA-mean	gene	Raw data	Mean SNP *χ* ^2^ (per gene)
MAGMA-top	gene	Raw data	Top SNP *χ* ^2^ (per gene)
MAGMA-pval	gene	SNP p-values, HapMap data	Mean SNP *χ* ^2^ (per gene)
MAGMA-pval-1K	gene, self-cont., comp.	SNP p-values, 1,000 Genomes data	Mean SNP *χ* ^2^ (per gene)
VEGAS	gene	SNP p-values, HapMap data	Mean SNP *χ* ^2^ (per gene)
PLINK-avg	gene, self-contained	Raw data	Mean SNP *χ* ^2^
PLINK-prune	gene, self-contained	Raw data	Mean SNP *χ* ^2^, SNP pruning
PLINK-top	gene	Raw data	Top SNP *χ* ^2^
ALIGATOR	competitive	SNP p-values	4 SNP p-value cut-offs
INRICH	competitive	SNP p-values	4 SNP p-value cut-offs
MAGENTA	competitive	SNP p-values	2 gene score quantile cut-offs

## Results

### Type 1 error rates

Simulation was used to assess the type 1 error rates, using permutations of the CD phenotype to obtain a global null distribution of no associated SNPs (see ‘Supplemental Methods—Simulation Studies’ for details). For the gene analysis, type 1 error rates were found to be controlled at the nominal level of 0.050 for the PC regression model, the summary statistics analysis model, as well as the SNP-wise models (Table S1 in S2 File).

The type 1 error rates for the gene-set analysis were also found to be well controlled for both the self-contained and competitive test (Table S2 in S2 File). For the competitive test an additional simulation using a polygenic null model was performed, with effects explaining a combined 50% of the phenotypic variance assigned to randomly selected SNPs. This polygenic type 1 error rate was also well controlled. The type 1 error rates for the self-contained analysis under the polygenic null model are also shown. These are considerably inflated because self-contained gene-set analysis by its definition is not designed to correct for polygenicity, illustrating the risk of performing self-contained analysis on polygenic phenotypes.

### Analysis of CD data—gene analysis

The results of the gene analyses of the CD data are summarized in Table 2, which shows the number of significant genes at a number of different p-value thresholds. Since the Type 1 error rates have been shown to be properly controlled these results can serve as a good indicator of the relative power of the different methods, and compared to simulation-based power estimates this has the advantage that no assumptions about the genetic causal model. From Table 2 it is clear that whereas the power of all the other methods is very similar, the MAGMA-main model shows a clear advantage over the rest. After Bonferroni correction, MAGMA-main found a total of 10 genome-wide significant genes, including the well-known CD genes *NOD2*, *ATG16L1* and *IL23R* [25,26]. This also indicates that although MAGMA can perform analysis of summary statistics, raw data analysis should always be preferred if possible.

**Table 2:** Number of significant genes at different p-value thresholds.

	P-value threshold					
Method	0.05	0.01	0.001	0.0001	Bonf.	Total genes
**Main analysis**						
MAGMA-main	1203	379	95	32	10	13172
MAGMA-mean	917	250	70	16	5	13172
MAGMA-top	934	244	61	16	5	13172
MAGMA-pval	927	241	64	16	5	12797
MAGMA-pval-1K	901	245	61	13	5	13075
PLINK-avg	944	239	56	16	4	13172
PLINK-top	903	242	64	13	5	13172
PLINK-prune	973	257	58	16	4	13172
VEGAS	915	225	61	17	6	12455
**Strat. correction**						
MAGMA-main	1141	352	89	28	8	13172
MAGMA-mean	897	240	62	14	4	13172
MAGMA-top	934	230	63	12	4	13172
**With 10kb Window**						
MAGMA-main	1611	505	126	45	13	16970
MAGMA-mean	1215	377	97	25	7	16970
MAGMA-top	1247	337	89	16	8	16970

‘Total genes’ gives the number of genes analysed. This was lower for the summary statistics analyses because some genes contained no SNPs present in both CD data and reference data and because VEGAS does not analyse the X chromosome. As such, those genes effectively have a p-value of 1 by default. For permutation-based methods, p-values were based on up to 1,000,000 permutations. No stratification correction was used in the analyses except the three under the ‘Strat. Correction’ header.

**Fig 1:**
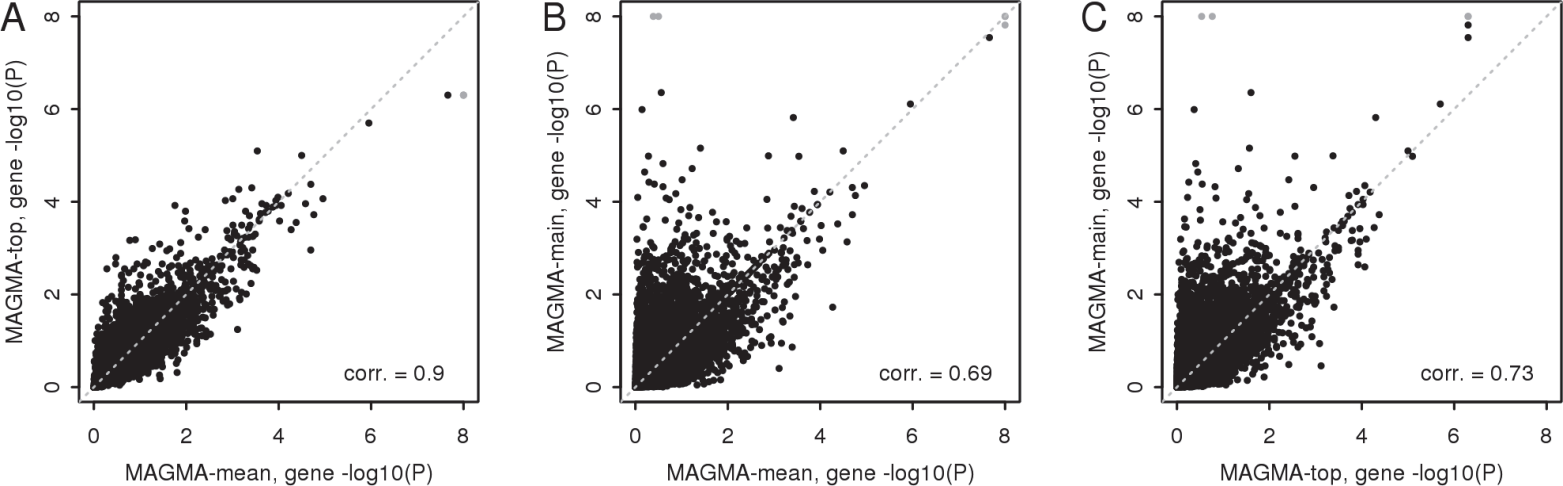
Comparison of gene analysis results for different test-statistics. Gene -log10 p-values from the CD data gene analysis in MAGMA for three different gene test-statistics, comparing analyses using (A) the mean *χ* ^2^ statistic with the top *χ* ^2^ statistic, (B) the mean *χ* ^2^ statistic and the PC regression model and (C) the top *χ* ^2^ statistic and the PC regression model. P-values below 10^–8^ are truncated to 10^–8^ (grey points) to preserve the visibility of the other points.

Specific implementation issues can be ruled out as the cause of the power difference since the PLINK and VEGAS analyses yield results highly similar to their matched MAGMA models (S9 Fig), and using the pruning option in PLINK also has little effect on the overall results. This means that the difference must be due to the difference in the methods and test-statistics themselves. Comparing the MAGMA implementations of these models in Fig 1, the mean *χ* ^2^ and top *χ* ^2^ approaches are shown to produce very similar p-values. Moreover, the plots reveal that the superior power of the MAGMA-main model does not arise from consistently lower gene p-values, but rather from a small set of genes with low p-values for MAGMA-main that are simply not picked up by the other approaches. This is likely to be related to the way LD between SNPs is handled, as that is one of the key differences between the multiple regression model of MAGMA-main and all the others. A post-hoc power simulation indeed indicates that multi-marker effects with weak marginals are the most probable explanation (see ‘Supplemental Methods—Simulation Studies’).

To increase the generalizability of these findings, two variations on the CD analyses were performed for MAGMA-main, MAGMA-mean and MAGMA-top. First, the analyses were repeated with 10 principal components computed from the whole data set as covariates to correct for possible stratification. The results are shown in Table 2 and S10 Fig. There is shown to be only very limited stratification, and although the power does decrease somewhat MAGMA-main’s power advantage is maintained. The analyses were also repeated with the gene annotation extended to include a 10 kilobase window around each gene, with the comparison in S11 Fig showing a considerable impact on the results. However, although this suggests that the choice of window can strongly affect the results of a gene analysis Table 2 shows that the relative power stays the same, with MAGMA-main again maintaining its superior power.

### Analysis of CD data—gene-set analysis

**Fig 2:**
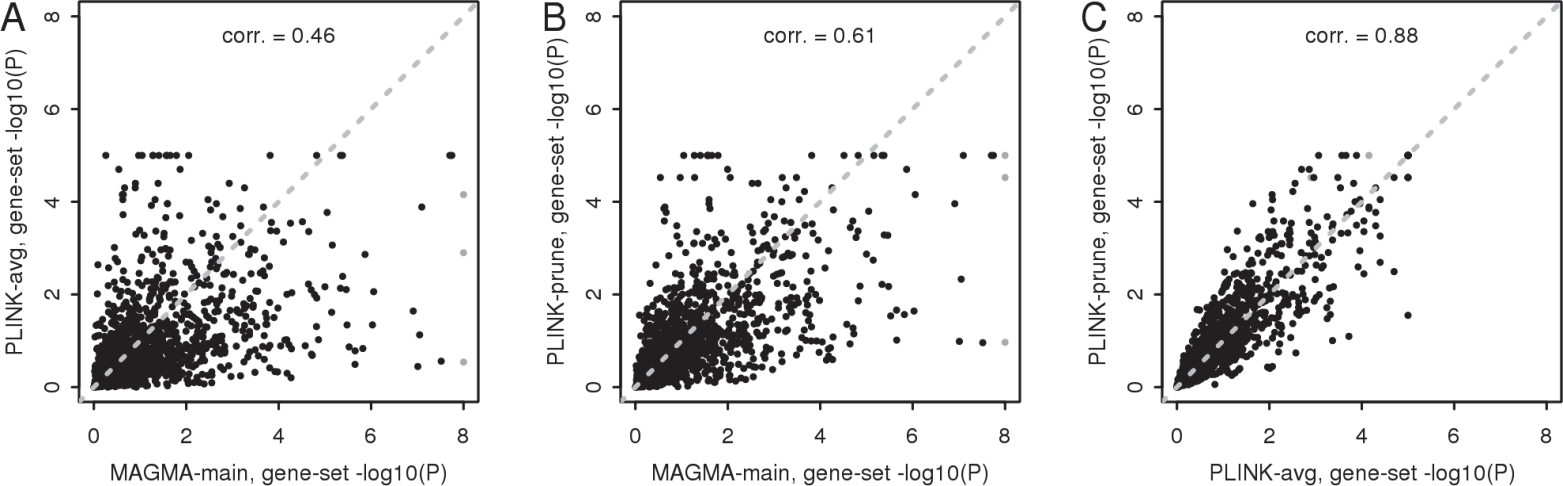
Comparison of self-contained gene-set analysis results. Gene set—log10 p-values from the CD data self-contained gene-set analysis for MAGMA and PLINK. Panel (A) shows the PLINK-avg (no pruning) results compared with the MAGMA-main analysis, panel (B) the PLINK-prune results compared with the MAGMA-main analysis and (C) the two PLINK analyses compared to each other. P-values below 10^–8^ are truncated to 10^–8^ (grey points) to preserve the visibility of the other points.

As with the gene analysis, the results of the CD analysis (Table 3 and Fig 2) can again serve as a gauge of the relative power of the different gene-set analysis methods. For the self-contained gene-set analysis this comparison is straightforward with MAGMA showing considerably more power than the two PLINK analyses. For the most part MAGMA’s power advantage can be explained by the difference in the underlying gene model, given the superior power of the PC regression model over the SNP-wise model used by PLINK shown before. Differences in how the genes are combined may also play a role however since, in contrast to PLINK, MAGMA weighs genes equally rather than by the number of SNPs in them and explicitly takes correlations between genes into account. Of note is also that PLINK-prune does considerably better than PLINK-avg, and that its p-values are somewhat more strongly correlated with those of the MAGMA analysis (Fig 2). An additional summary statistics analysis (MAGMA-pval-1K) on SNP p-values and using 1,000 Genomes reference data was also performed. This showed less power than PLINK even though it uses the same model at the gene level, suggesting that the difference is due to how the genes are aggregated to gene-sets. One of the key differences in this regard is that PLINK gives larger genes greater weight whereas MAGMA weighs them equally. As such a likely explanation is that the PLINK results are partially driven by a smaller number of large genes, though constructing the intermediate models to verify this is beyond the scope of this paper.

**Table 3:** Number of significant gene sets at different p-value thresholds.

	P-value threshold				
Method	0.05	0.01	0.001	FWER	Tested gene sets
**Self-contained**					
MAGMA-main	448	253	120	39	1320
MAGMA-pval-1K	257	108	28	4	1320
PLINK-avg	329	160	67	19	1320
PLINK-prune	361	181	86	27	1320
**Competitive**					
MAGMA-main	85	25	9	1	1320
MAGMA-main (no size correction) ^a^	105	33	9	3	1320
MAGMA-pval-1K	80	11	3	1	1320
ALIGATOR (cut-off = 0.01)	94	38	12	0	653
ALIGATOR (cut-off = 0.005)	85	23	7	0	508
ALIGATOR (cut-off = 0.001)	59	34	10	0	149
ALIGATOR (cut-off = 0.0001)	28	24	6	0	35
INRICH (cut-off = 0.01)	79	22	3	0	777
INRICH (cut-off = 0.005)	74	23	7	0	602
INRICH (cut-off = 0.001)	66	39	15	0	213
INRICH (cut-off = 0.0001)	41	22	8	3	57
MAGENTA (cut-off = 5^th^ quant.)	83	20	4	0	952
MAGENTA (cut-off = 1^st^ quant.)	50	25	6	0	389

The FWER column corresponds to p-values below 0.05 after family-wise error correction, using Bonferroni correction for MAGMA, PLINK and MAGENTA and built-in FWER methods for INRICH and ALIGATOR. The ‘Tested gene sets’ column shows the number of gene sets for which p-values were computed, which were lower for INRICH, ALIGATOR and MAGENTA because some gene sets contained insufficiently many SNPs/intervals/genes with p-value below the chosen cut-off. Note that such gene sets do remain part of the analysis and count towards the total number of tests conducted, their p-values are effectively set to 1.

^a^ in this analysis the default correction for gene size and gene density was turned off

The comparison of competitive methods is somewhat more complicated, due to the fact that ALIGATOR, INRICH and MAGENTA all use discretization using a p-value cut-off. This cut-off needs to be specified by the user and has no obvious default value, although for MAGENTA the 5^th^ percentile cut-off is suggested as the most optimal [12]. For ALIGATOR and INRICH the analysis was therefore performed at four different cut-offs (0.0001, 0.001, 0.005, 0.01), and for MAGENTA at two (5^th^ and 1^st^ percentile).

Of the four tools, only MAGMA and INRICH yield significant results after multiple testing correction (Tables 3 and 4). As with the self-contained gene-set analysis, power for the MAGMA analysis is better when using raw data rather than SNP p-values as input, though both yield one significant gene set. For INRICH the results are strongly dependent on the SNP p-value cut-off used, with three significant gene sets at the 0.0001 cut-off but none at the higher ones, further emphasizing the problem of choosing the correct cut-off. It should also be noted that the p-values have not been corrected for the fact that the gene-sets have been analysed under four different thresholds, and thus might not fall below the significance threshold if they were.

**Table 4:** Competitive gene-set p-values for MAGMA and INRICH significant gene-sets.

	MAGMA-main		MAGMA-pval	INRICH	
Gene-set	Size correction	No correction		Cut-off = 0.0001	Cut-off = 0.01
Regulation of AMPK activity via LKB1	**0.000026**	**0.000022**	0.059	1^a^	0.37
ECM receptor interaction	0.000094	**0.000015**	0.00052	1^a^	0.08
Cell adhesion molecules	0.0001	**0.000011**	0.012	1^a^	0.11
Cytokine receptor interaction	0.004	0.01	**0.000028**	0.0007	0.091
TCR calcium pathway	0.034	0.024	0.11	**0.0001**	0.074
NKT pathway	0.052	0.073	0.034	**0.0001**	0.0022
IL27 pathway	0.3	0.36	0.22	**0.0001**	0.123

Significant p-values are highlighted in bold. MAGMA p-values compared against a Bonferroni-corrected threshold of 0.05/1320 = 0.000038. For INRICH, corrected p-values (not shown) are compared against a threshold of 0.05; corrected p-value for all three significant gene-sets is 0.049.

^a^ p-values were not computed because fewer than two genes in the set overlapped with an associated interval; p-values are therefore effectively equal to 1

**Fig 3:**
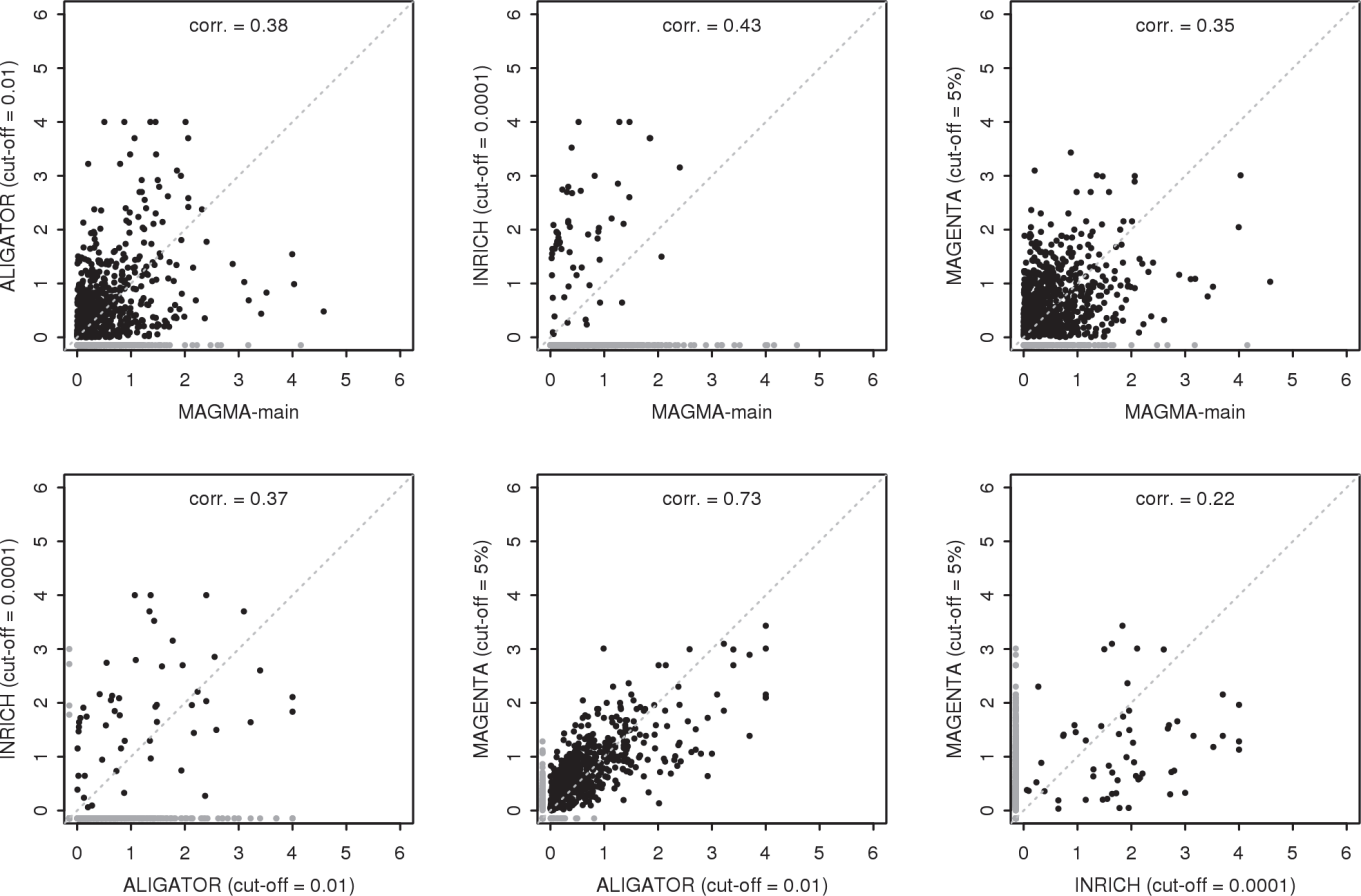
Comparison of competitive gene-set analysis results. Gene set -log10 p-values from the CD data competitive gene-set analysis for MAGMA, ALIGATOR, INRICH and MAGENTA. Results for ALIGATOR and INRICH are shown for each for the SNP p-value cutoff that yielded the highest observed power (0.01 and 0.0001 respectively), MAGENTA at the advised 5^th^ percentile cutoff. P-values for gene sets not evaluated by one of the methods are shown in grey. The shown correlations are for the -log10 p-values for gene-sets evaluated by both methods.

**Fig 4:**
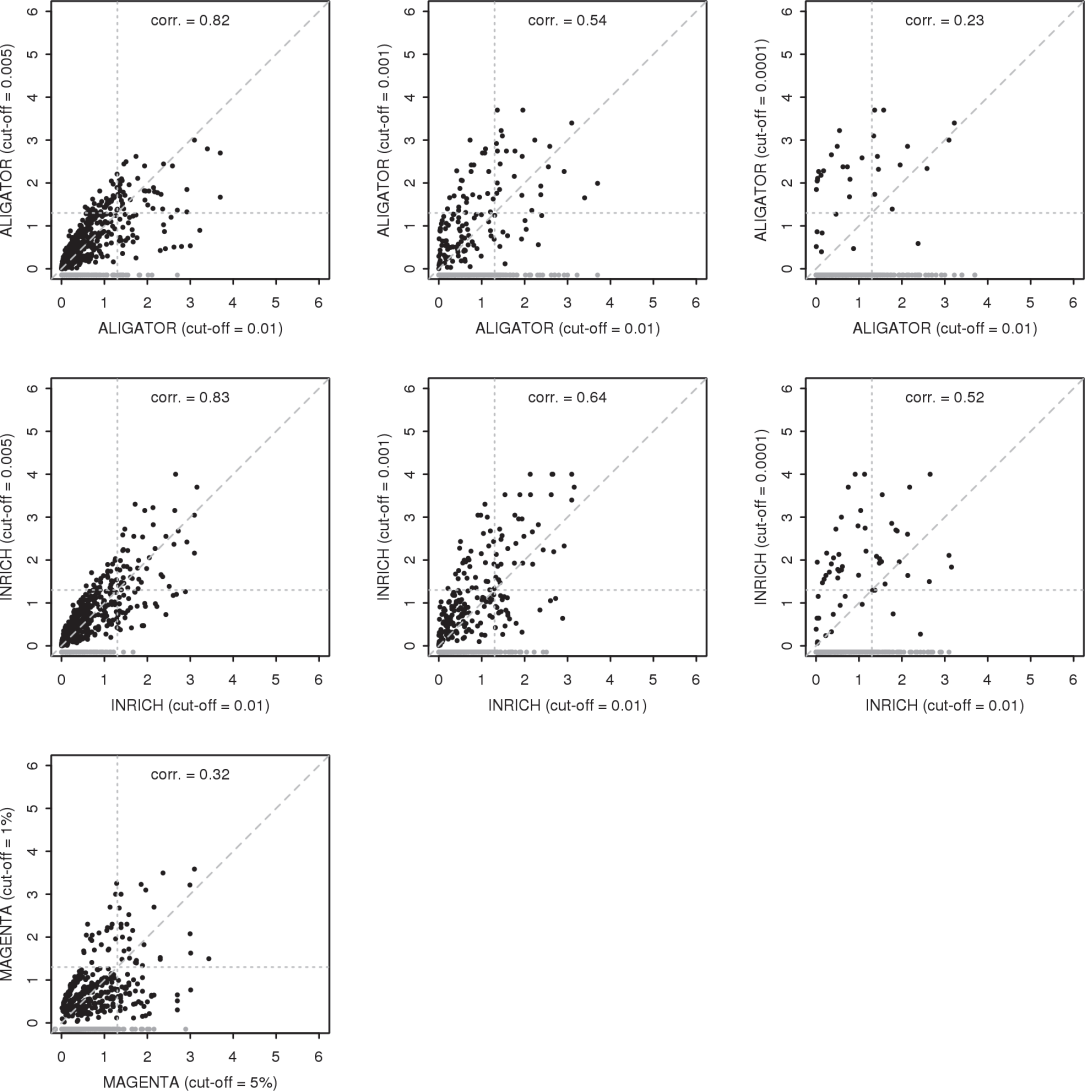
Comparison of competitive gene-set analysis results at different SNP cut-offs. Comparison of gene set -log10 p-values from the CD data competitive gene-set analysis at different SNP p-value cut-offs for ALIGATOR (top row), INRICH (middle row) and MAGENTA (bottom row). The highest cut-off on the horizontal axis is compared to each of the lower cut-offs. P-values for gene sets not evaluated at the lower cut-off are shown in grey. The shown correlations are for the -log10 p-values for gene-sets evaluated at both cut-offs. Horizontal and vertical grey dotted lines demarcate the p = 0.05 nominal significance threshold.

Looking at the results in more detail (Fig 3) also suggests that the differences in results are not merely due to a difference in power. The concordance between methods is poor, with only MAGENTA and ALIGATOR showing a reasonable correlation in results. Moreover, there is considerable discordance between different p-values cut-offs for the same methods as well (Fig 4). This suggests that the different methods, or methods at different p-value cut-offs, are sensitive to distinctly different kinds of gene set associations. In particular, MAGMA and the other three methods at higher p-value cut-offs would be expected to respond best to gene-sets containing a larger number of somewhat associated genes. Conversely, at lower p-value cut-offs the latter three should become more sensitive to gene-sets containing a small number of more strongly associated genes. This is exemplified by the INRICH analysis. At the 0.0001 cut-off only quite strongly associated genes are counted as relevant, but as there are only 42 such genes overall the three gene sets (containing either 26 or 29 genes) become significant despite each containing only three relevant genes.

Aside from differences between methods, Table 3 also shows a clear difference between self-contained and competitive gene-set analysis. This is not a difference in power, but rather a difference of null hypothesis. Competitive tests attempt to correct for the baseline level of association present in the data and accordingly have a much more general null hypothesis. The impact of this difference in hypothesis can be illustrated by comparing the MAGMA self-contained and competitive analyses, since they are performed in the same framework. Whereas the self-contained analysis detects 39 gene sets that show association with the phenotype, the competitive analysis detects only one of those 39. For the remaining 38 gene sets, there is no evidence in the data that the associations in those gene sets are any stronger than would be expected by chance given the polygenic nature of CD. The gene-set that remains is the *Regulation of AMPK via LKB1* (REACTOME) set. For two additional gene sets, *Cell Adhesion Molecules* (KEGG) and *ECM-receptor Interaction* (KEGG), the competitive p-value also drops below the significance threshold (Table 4 and S12 Fig) if the correction for gene size and gene density is turned off. This suggests that these gene sets do in fact contain significantly elevated levels of association, but that this is partially caused by confounding effects of the size and density of the genes they contain. Given the strength of the confounding effect it is evident that gene-set analyses should always be corrected for these and other potential confounders, to avoid false positive results. Full results for the analyses can be found in Table S5 in S2 File.

### Computational performance

All analyses were performed on the Genetic Cluster Computer, which is part of the Dutch Lisa Cluster. In terms of computational performance MAGMA is shown to have a considerable advantage over the other methods (Table 5) for both gene and gene-set analysis. The most marked difference is between MAGMA and PLINK, the only one of the alternative methods using raw data input. However, the raw data analysis in MAGMA outperforms the summary statistics methods as well. Although INRICH and ALIGATOR show comparable computation times at their lowest SNP p-value cut-off, the need to repeat the analysis at multiple cut-offs means the total analysis for both takes considerably longer.

**Table 5:** Computation times for gene and gene-set analyses.

Method	Computation time	Factor	Type
**Gene analysis**			
MAGMA-main	00:00:44	1	Raw data
MAGMA-mean	00:01:00	1.4	Raw data
MAGMA-top^a^	00:25:18	34.5	Raw data
MAGMA-pval	00:00:10	0.3	Summary
MAGMA-pval-1K	00:00:54	1.2	Summary
PLINK-avg^b^	11:35:05	947.8	Raw data
PLINK-prune^b^	08:55:13	729.8	Raw data
PLINK-top^b^	10:59:26	899.2	Raw data
VEGAS^a^	03:14:05	264.7	Summary
MAGMA-main (10 covariates) ^c^	00:00:58	1.3	Raw data
PLINK-avg (1 covariate)^c^ ^,^ ^d^	160:39:03	13144.2	Raw data
PLINK-avg (10 covariates)^c^ ^,^ ^d^ ^,^ ^e^	> 857:54:57	> 70193.1	Raw data
**Gene-set analysis**			
MAGMA-main	00:01:56	1	Raw data
MAGMA-pval-1K	00:01:09	0.6	Summary
PLINK-avg^b^	44:20:40	1376.2	Raw data
PLINK-prune^b^	62:35:24	1942.4	Raw data
ALIGATOR total (4 cut-offs)^f^	02:37:11	81.3	Summary
Cut-off = 0.01	01:23:15	43.1	Summary
Cut-off = 0.0001	00:07:54	4.1	Summary
INRICH total (4 cut-offs)^g^	01:09:22	35.9	Summary
Cut-off = 0.01	00:33:41	17.4	Summary
Cut-off = 0.0001	00:05:16	2.7	Summary
MAGENTA	00:24:35	12.7	Summary

‘Factor’ indicates the increase in computation time relative to MAGMA-main. MAGMA computation times for gene-set analysis include both self-contained and competitive tests. All analyses were run on the same system.

^a^ up to 100,000 permutations

^b^ up to 10,000 permutations

^c^ covariates are PCs used for stratification correction

^d^ 1,000 permutations

^e^ did not complete

^f^ 5,000 permutations, 1,000 replications

^g^ 10,000 replicates, 10,000 bootstraps

The low MAGMA computation times are largely due to the choice of statistical model. Since the statistical tests used have known asymptotic sampling distributions the need for computationally demanding permutation or simulation schemes is avoided. Note however that the permutation-based SNP-wise analyses in MAGMA also show very reasonable computation times. These results demonstrate that, given efficient implementation, there is no computational reason to prefer analysis of summary statistics over raw data analysis, even when using permutation.

## Discussion

We have developed MAGMA, a fast and flexible method for performing gene and gene-set analysis in a two-tiered parametric framework. Comparison with a number of other, frequently used methods shows that MAGMA has better power for gene analysis as well as for both self-contained and competitive gene-set analysis. An important factor in this is the multiple regression model used in the gene analysis, which is better able to incorporate the LD between SNPs than other methods. Because of its two-layer structure, this improvement in power at the gene-level subsequently carries over to the gene-set analysis.

MAGMA was also found to be generally much faster than other methods, even methods that used only summary statistics rather than raw data. This is primarily due to the choice of statistical model, which did not require the kind of computationally expensive permutation or sampling procedures used in the other methods. However, even the permutation-based SNP-wise models implemented in MAGMA outperformed their equivalents in other software and yielded very reasonable computation times.

Although MAGMA showed better power than other tools for both the self-contained and competitive gene-set analysis, these comparisons also revealed considerable differences between the methods. This was most pronounced for the competitive gene-set analysis, with even results for individual methods showing significant variability based on the choice of cut-off. At present no comprehensive evaluation of the differences between existing gene-set analysis methods exists, leaving the causes and implications of these difference unclear. It is beyond the scope of this paper to perform such an evaluation, but the degree of discordance between most methods strongly suggests a need for future research in this direction. An additional caveat is that it is unknown to what extent the observed differences in power between methods may depend on the specific genetic architecture of Crohn’s diseases, and as such generalizing the results to other genetic architectures must be done with caution.

The framework for MAGMA is built with future extensions in mind. Because of the two-tiered structure of the gene-set analysis, alternative gene analysis models are straightforward to implement and are automatically available for use in the gene-set analysis. Similarly, the linear regression structure used to implement the gene-set analysis offers a high degree of extensibility. At present it enables analysis of continuous gene-level covariates as well as conditional analysis of gene-sets correcting for possible confounders, and the analysis of the CD data demonstrates that correction for confounders such as gene size and gene density is indeed strongly advised. The model is easily generalized to much more general gene-level linear regression models to allow for simultaneous analysis of multiple covariates and gene-sets, opening up a wide range of new testable hypotheses.

## Supporting Information

S1 File Supplemental methods. (PDF)

S2 File Supplemental tables. (PDF)

S3 File Results for all Crohn’s Disease gene and gene-set analyses. (XLSX)

S1 Fig Validation of F-test for binary phenotypes. Empirical p-values were obtained for the CD data PC regression gene analysis by permutation of the F-statistic (A), in order to verify the accuracy of the asymptotic F-test p-values. An initial 100,000 permutations were computed for each gene. For genes with a very low initial empirical p-value (shown in blue and red) the number of permutations was increased to about 500 million to refine the empirical p-value. The dashed horizontal line indicates the lowest possible non-zero permutation p-value, genes with an empirical p-value of 0 are shown at half that minimum p-value in the plot (in red). The process was repeated using a subsample of the CD data skewed 4:1 towards cases (B) or controls (C); and with evenly divided subsamples of N = 1000 (D), N = 500 and N = 250. Only the initial 100,000 permutations were performed for these analyses, genes with an empirical p-value of 0 are again shown at half the minimum non-zero p-value (in blue).
(TIFF)

S2 Fig Comparison of linear and logistic model. Gene p-values were computed using a logistic regression model to compare against the linear regression model used in MAGMA. P-values were computed using either a Score test (A) or a Likelihood Ratio test (B). Because the Likelihood Ratio test appeared to have significantly more power than both the Score test and the MAGMA F-test, empirical p-values for the Likelihood Ratio test were computed by generating up to 10,000 permutations of the Likelihood Ratio statistic. This was compared to the asymptotic Likelihood Ratio test p-values (C), revealing a downward bias in the asymptotic p-values. The empirical p-values were then compared to the MAGMA F-test p-values (D), which shows that the apparent power advantage of the Likelihood Ratio test in (B) was due to the bias in the p-values.
(TIFF)

S3 Fig The effect of genotype pruning. The pruning implemented in MAGMA was applied to the genes in the CD data at different levels of the prune factor *f* (default is 0.999), which reflects the proportion of the total variance in the raw genotype data that is retained after pruning. The original number of genotyped SNPs in each gene is plotted against the number of PCs retained after pruning. The regression slope gives an estimate of the average proportion of PCs to SNPs.
(TIFF)

S4 Fig Comparison of pruning to PLINK independent SNPs. The PLINK—indep option was used to obtain an estimate of the number of independent SNPs at different *R* ^*2*^ values. The number of PCs retained by MAGMA at different values of the pruning factor *f* is plotted against the number of independent SNPs at the *R* ^*2*^ value that provided the closest match.
(TIFF)

S5 Fig Evaluation of the genotype imputation procedure. MAGMA needs to impute missing genotype values in order to run the multiple regression model, which is done by single imputation using flanking SNPs. To validate this procedure a subset of genes was selected from the CD data, and genotype values in those genes were set to be missing for a specified fraction of all the genotype values (up to 10%), and gene p-values were then computed after using the imputation to fill in those missing values. Gene p-values were also computed for the original full data. For each fraction, missing data was simulated 100 times for each gene, and the 5^th^ (black) and 95^th^ (blue) quantiles of the p-values of each gene were computed and plotted against that gene’s full data p-value.
(TIFF)

S6 Fig Distribution of correlations between gene Z-statistics. Gene analysis was performed on the CD data, and a joint empirical distribution gene SSM values was generated using 4,611 permutations of the phenotype (since the sample size of the CD data is 4,611). The correlation matrix was then computed from this distribution. In addition, a correlation matrix for 13,172 uncorrelated genes was simulated by generating 4,611 permations for 13,172 genes and computing the correlation matrix. This provides the distribution of correlation coefficients that would be expected if the genes were uncorrelated. A QQ-plot of these expected correlation coefficients are plotted against the observed correlation coefficients in (A), showing a clear surplus of high positive correlations for the CD data genes. A QQ-plot using only correlations between genes more than 5 megabases apart (B) reveals that this is due to short-range correlations only.
(TIFF)

S7 Fig Visualisation of the gene Z-statistic correlation matrix for chromosomes 5 and 6. Gene analysis was performed on the CD data, and a joint empirical distribution of the gene SSM values was generated using 4,611 permutations of the phenotype (since the sample size of the CD data is 4,611). The correlation matrix for chromosomes 5 and 6 was plotted, with individual pixels corresponding to a pair of genes and the color (from white to black) proportional to the absolute value of the correlation between those genes. Correlations with absolute value smaller than 0.05 are set to 0 to reduce noise. The yellow area corresponds to genes within 5 megabases of each other, corresponding to gene pairs for which MAGMA computes the correlations (correlations between more distant genes are assumed to be 0); the dashed lines indicate the boundary between the two chromosomes.
(TIFF)

S8 Fig Quality of reference data-sets for summary statistics gene analysis. Summary statistics gene analysis of CD data SNP p-values was performed using different reference data-sets, using the SNP-wise mean *χ* ^2^ model. This was compared to the same SNP-wise analysis performed on the raw CD genotype data. Grey points correspond to genes not covered by the reference data-set. The reference data-sets used are (A) the CD data itself, (B) the 1,000 Genomes European panel (97 missing genes), (C) the HapMap 3 European panel (375 missing genes) and (D) the HapMap 3 African panel (623 missing genes).
(TIFF)

S9 Fig Comparison of VEGAS and PLINK with matched MAGMA SNP-wise models. Gene -log10 p-values from the CD data gene analysis for equivalent gene test-statistics implemented in different tools. The gene test-statistics used are (A) the mean *χ* ^2^ statistic in MAGMA and PLINK, (B) the top *χ* ^2^ statistic in MAGMA and PLINK, (C) the mean *χ* ^2^ statistic in MAGMA and VEGAS with analysis based on SNP p-values and HapMap 3 reference data and (D) the mean *χ* ^2^ statistic in MAGMA on raw data and with analysis based on SNP p-values and HapMap 3 reference data.
(TIFF)

S10 Fig Comparison of MAGMA gene analysis models with and without PCs as covariates. Gene -log10 p-values from the CD data gene analysis for the three MAGMA gene analysis models with 10 PCs as covariates to correct for stratification, and without. P-values below 10^–8^ are truncated to 10^–8^ (grey points) to preserve the visibility of the other points.
(TIFF)

S11 Fig Comparison of MAGMA gene analysis models with and without 10kb window. Gene -log10 p-values from the CD data gene analysis for the three MAGMA gene analysis models with additional 10 kilobase window around the transcription start and stop sites, and without. Genes only present in the 10 kilobase window analyses are omitted. P-values below 10^–8^ are truncated to 10^–8^ (grey points) to preserve the visibility of the other points.
(TIFF)

S12 Fig Comparison of MAGMA competitive gene analysis with and without correction for gene size and gene density. Gene -log10 p-values from the CD data analyses. When the correction is turned on (the default setting), the gene-set effect is conditioned on gene size and gene density. Grey dashed lines represent the Bonferroni-corrected significance threshold. The effective size of the gene (number of PCs in the gene after pruning) is used as a measure of gene size, the ratio of effective size and total number of SNPs as a measure of gene density. The correction is achieved by entering gene size and gene density, as well as the log of both, as predictors in the generalized gene-set analysis model alongside the gene-set indicator variable.
(TIFF)
