# Supplementary material for: MAGMA: Generalized Gene-Set Analysis of GWAS Data
Source: PLoS Comput Biol. 2015 Apr 17;11(4):e1004219. doi: 10.1371/journal.pcbi.1004219 (PMC4401657; doi:10.1371/journal.pcbi.1004219)
Supplement: S1 File — (PDF) [file pcbi.1004219.s001.pdf]

Supplemental methods

MAGMA: Generalized Gene-set Analysis of GWAS Data

Christiaan A. de Leeuw,[1,2,*] Joris M. Mooij,[4] Tom Heskes,[2] Danielle Posthuma[1,3]

[1] Department of Complex Trait Genetics, VU University Medical Center/VU University Amsterdam, Amsterdam, The Netherlands

[2] Institute for Computing and Information Sciences, Radboud University Nijmegen, Nijmegen, The Netherlands

[3] Department of Clinical Genetics, VU University Medical Centre Amsterdam, Neuroscience Campus Amsterdam, The Netherlands

[4] Informatics Institute, University of Amsterdam, Amsterdam, The Netherlands

*Corresponding author
E-mail: c.a.deleeuw@vu.nl (CL)

Simulation studies

Type 1 error rates

Type 1 error rates for both gene and gene-set analysis were assessed under a global null model by analysing 1,000 permutations of the CD phenotype, and computing the proportion of p-values smaller than 0.05. In addition to the MSigDB gene-sets, for the gene-set analysis additional simulated gene-sets were used to more systematically study the effect of correlation between genes on type 1 error rates. To do so, gene-sets were generated by randomly sampling batches of adjacent genes from the data, increasing the size of those batches to increase the correlation between genes. Gene-sets of 10 and of 100 genes were generated using batch sizes of one, two or five, for a total of six conditions. For each condition 1,000 gene-sets were simulated.

The global null model used in this type 1 error simulation implies that none of the genes in the data are associated with the phenotype, which corresponds to the null hypotheses of the gene analysis and the self-contained gene-set analysis. However, the competitive null hypothesis makes no such assumption. The competitive gene-set analysis is designed to correct for background levels of association and should maintain its nominal type 1 error under any polygenic model, as long as the subset of SNPs and genes associated with the phenotypes is not related to the gene-sets. As such a second type 1 error simulation was conducted for the competitive gene-set analysis, using such a polygenic null model.

For this polygenic type 1 error simulation, to simulate phenotypes 1,000 SNPs were randomly selected from the CD data and normalized. Effect sizes for each SNP were drawn from a standard normal distribution, and a genetic effect was computed as the sum of the SNP genotypes weighted by these effect sizes. The simulated phenotype was then obtained by adding normally distributed noise to this genetic effect, with the variance of the noise distribution chosen such that the phenotypic variance explained by the genetic effect (ie. the chip-heritability) was 50%. As with the first type 1 simulation, this procedure was repeated 1,000 to generate 1,000 simulated phenotypes were generated, and the type 1 error rate was computed for each gene-set as the proportion of those 1,000 simulations for which its p-value was smaller than 0.05.

For the gene analysis, the type 1 error rates averaged over all genes are shown for the different gene test-statistics in Table S1. In addition to those for the test-statistics used in the CD analysis discussed in the paper, error rates for gene analysis using the unweighted (MAGMA-unwZ) and weighted (MAGMA-wtdZ) mean *Z* test-statistics are shown as well. As described in the 'SNP-wise gene analysis' section below, for these test-statistics a normal distribution is fitted to the empirical sampling

distribution, which is used to compute the p-value. Although this results in slightly elevated type 1 error rates they are still within reasonable limits.

The type 1 error rates for the gene-set analysis are shown in Table S2. The middle two columns show error rates for the estimates under the global null model; the last two columns shows the error rates under the polygenic null model. The global type 1 error rate for the self-contained test and both type 1 error rates for the competitive test are found to be well-controlled for both the MSigDB and the simulated gene-sets. The polygenic type 1 error rates for the self-contained gene-set analysis are shown as well, to illustrate the risk of using self-contained tests for polygenic phenotypes.

Under a polygenic model, the expected degree of association with the phenotype of a random gene is greater than it would be under the global null hypothesis. Consequently, any gene set is likely contain some amount of association with the phenotype by chance, with this amount increasing as gene sets get larger, with lower self-contained p-values as a result. In other words, there is some probability that for an arbitrary gene set the self-contained null hypothesis is simply not true. Although this is technically not a type 1 error, it does mean that for a polygenic phenotype the self-contained p-value will at least in part simply reflect the size (and other properties) of the gene set rather than any genuine biological relevance.

Post hoc power simulations for gene analysis

For the gene analysis, to gain more insight into the difference in power between the multiple regression model and the more traditional SNP-wise methods, two targeted power simulations were conducted. The goal of the first simulation was to study the dependence of power on local LD, an issue previously demonstrated by Moskvina et al.[14]. The second simulation was performed to study differences between methods in the power to detect obscured multi-marker effects.

In both simulations normally distributed phenotypes were simulated with a population explained variance of either 0.1% or 0.5%. Ten genes, each containing five SNPs, were randomly selected from the CD data. A total of 1,000 phenotypes were generated for each gene in each condition, and the power averaged over these genes was computed per condition. The SNPs were standardized prior to generating the simulated effect, to eliminate differences in MAF.

In each simulation, a genetic effect as a function of the SNPs in the gene was defined (as described below), and then a normally distributed noise term was added to that genetic effect to obtain the desired degree of population explained variance. This was accomplished by choosing the variance σ_e^2 of the the noise distribution such that $\sigma_g^2 / (\sigma_g^2 + \sigma_e^2) = R_{\text{pop}}^2$, with σ_g^2 the variance of the genetic effect and R_{pop}^2 the desired population explained variance.

For the first simulation, the middle SNP of each gene was designated the 'high LD' SNP. A copy of the high LD SNP was then added to the gene, which was subsequently permuted to remove its LD with the other SNPs and designated the 'low LD' SNP. Phenotypes were simulated with effects for either the high LD or the low LD SNP. Since only one effect SNP was used each time, the genetic effect for each individual was thus defined simply as the value for that SNP designated

For the second simulation two positively correlated SNPs in each gene were designated as effect SNPs. These were assigned effect sizes of equal magnitude but opposite in direction, to create a multi-marker effect with relatively small marginal effects for the individual markers. Thus, the genetic effect for each individual was defined as the difference between the values of the two effect SNPs for that individual.

The simulations were analysed using the main MAGMA gene analysis model as well as the SNP-wise MAGMA-chi and MAGMA-top models. Given the equivalence of the latter with PLINK and VEGAS, the results of these simulations generalize to those tools as well. These results are shown in Table S3.

The first simulation demonstrates the effect of LD around associated SNPs. For MAGMA-main there is no effect of LD, with power depending only on the effect size. Similarly, because it only uses the most strongly associated SNP and not its neighbors, MAGMA-top is also mostly unaffected. For MAGMA-chi however the results are strongly influenced by the LD surrounding the associated SNP, with power skewed towards high LD SNPs at the expense of low LD SNPs.

The second power simulation demonstrates obscured multi-marker effects, and it is clear from the results that only the multiple regression model can properly detect such effects. The two SNP-wise models show markedly lower power, and further inspection of the indivual genes reveals that this is also highly variable across genes (Table S4). Whereas power for some genes is only somewhat impaired, it drops to the chance level of 0.05 for others.

Although due to LD one would typically expect the effects of pairs of positively correlated SNPs to have the same direction, the opposite are possible as well. It might occur for example in case of a haplotype effect or when a causal SNP not present in the data is poorly marked by genotyped SNPs, which would manifest as joint effects of correlated SNPs with weak marginal associations for each SNP individually. Moreover, the results of the CD analysis suggest that these in fact do occur, as the multiple regression model displays significantly higher power. Since a key difference between this model and the two SNP-wise methods lies in the different way of handling LD, it follows that this power difference is likely related to LD. The good performance of MAGMA-top in the first simulation indicates that it is not due to the effect described by Moskvina et al[14]., making multi-marker effects the most viable culprit. This is also supported by a closer inspection of the MAGMA-main top genes not picked up by the SNP-wise models. Among these genes the percentage of SNPs with p-values

below 0.01 is only 2%, which is barely higher than in the data-set as a whole and far below the 66% in the overall top genes.

Implementational details of the MAGMA model

Gene analysis for binary phenotypes

For binary phenotypes MAGMA uses the same linear model as used for continuous phenotypes, resulting in a so-called linear probability model. This linear model is considerably faster to run than more traditional logistic regression model, but it does violate a number of assumptions of the linear regression model and the F-test (normally distributed errors, linearity, homoscedasticity). To verify that this does not bias the resulting p-values, the asymptotic p-values from the F-test on the CD data are compared to empirical p-values. These empirical p-values are obtained by permuting the CD phenotype to generate an empirical sampling distribution for the F-statistic. The p-values are then computed as the proportion of permutations for which the F-statistic is greater than the observed F-statistic.

An initial 100,000 permutations were used for each gene in the CD data. This was subsequently increased to about 500 million permutations for genes with a very low empirical p-value. As the results in Figure S1a show, the asymptotic p-values are essentially identical to the permuted p-values, indicating that despite the violated assumptions the resulting p-values are still valid. Only for four very low p-values did the asymptotic and empirical p-values diverge, since the number of permutations was insufficient to obtain empirical p-values as low as those of the F-test (the empirical p-values were all 0). Since the p-values matched for all other genes however, it can reasonably be assumed that this applies to these four genes as well if sufficiently many additional permutations were computed.

Because the CD phenotype has a roughly even distribution of cases and controls, the process was repeated for two skewed subsamples of the CD data. The subsamples were obtained by randomly dropping either controls (or cases) from the data until a 4:1 (or 1:4) case:control ratio was obtained. For these subsamples only 100,000 permutations were used. The results in Figure S1b and S1c show that the asymptotic p-values are still valid. Similarly, the analysis was repeated on small subsamples of the CD data down to a sample size of 250 (Figure S1d through S1f). These plots show that the asymptotic p-values become slightly more conservative but are otherwise valid in this case as well.

To determine whether the use of a linear rather than a logistic model affected the detection of gene associations, the F-test p-values were also compared to p-values based on a logistic regression model using either the Score test or the Likelihood Ratio test (both computed using R). These are shown in Figure S2a and S2b. The Score test yields p-values virtually identical to those of the F-test. On the other hand, the Likelihood ratio test appears to have more power, with lower p-values than the F-test for 66.4% of the genes. This appears to be due to a downward bias in the

Likelihood Ratio test p-values however, as a comparison of those p-values with empirical p-values based on permutation of the Likelihood Ratio statistic reveals (Figure S2c). When the F-test p-values are compared to these empirical Likelihood Ratio p-values (Figure S2d), no substantive evidence for a difference in power remains. This suggests that no relevant information is lost when using a linear rather than a logistic model.

Pruning genotype data

The degree of pruning performed prior to running the gene analysis is controlled by the pruning parameter *f*, which reflects the proportion of variance to retain from the original genotype data matrix for a gene. With λ_i the eigenvalue for the *i*th principal component and *K* the number of SNPs in a gene, the total variance is given as $V_{\text{tot}} = \sum_i^K \lambda_i$. With the principal components sorted in descending order of their eigenvalue the variance retained in a subset of the first *j* principal components is $V_j = \sum_i^j \lambda_i$. When pruning, the smallest *j* such that $V_j / V_{\text{tot}} \geq f$ is selected. By default $f = 0.999$, retaining all but 0.1% of the total variance in the gene. However, due to the high LD between neighbouring SNPs this will still tend to result in a significant decrease in the number of PCs relative to *K*, as shown in Figure S3. Alternatively the degree of pruning can also be controlled by specifying the number of PCs to retain either as a fixed number or as a proportion of the number of the number of SNPs in a gene. In this case MAGMA will still maintain the upper bound *f* to ensure identifiability of the regression model.

Imputation of missing genotype data

Even with the most stringent quality control procedures almost any GWAS data-set will contain a small amount of missing genotype values. This presents a problem for the PC regression model since it requires a complete genotype data matrix to work, and unlike with single-marker analysis listwise deletion of individuals with missing genotypes is not a viable option. As such a single imputation approach is used to fill in these missing values. Although typically a multiple imputation approach would be preferred because it gives somewhat more stable results, this would also complicate the model and thus lead to longer computation times. Moreover, since in practice the missing will tend to be very low the difference between single imputation and multiple imputation should be minimal.

It should be noted that the procedure used here is a *statistical imputation* procedure, and should not be confused with the kind of *genotype imputation* procedure implemented in programs like MACH and IMPUTE. The former is a standard statistical technique aimed at filling in missing values in partially observed variables using the observed data. The latter is aimed primarily at filling

in entirely unobserved variables using the observed data in combination with an external reference data-set with a sufficiently similar LD structure as the source population of the original data-set. Although in principle genotype imputation can also be used to fill in missing values in partially observed variables this is unlikely to lead to better results than those of conventional statistical imputation techniques, as the reference data-set will never fully match the LD structure in the observed data. Moreover, since the missing values in the genotype data are essentially just a nuisance problem and the computation time of genotype imputation is orders of magnitude longer than that of statistical imputation, the latter should be preferred.

The imputation implemented in MAGMA is as follows. For each SNP *j* with missing data, two linear probability models (LPM) are fitted, using up to five flanking SNPs on each side of *j* as predictors. The first LPM (LPM1) uses the whole sample to predict whether an individual *i* is a homozygote for the major allele or not ($x_{ji} = 0$). The second LPM (LPM2) uses all individuals who are not a homozygote for the major allele to predict whether an individual is a heterozygote ($x_{ji} = 1$) or a homozygote for the minor allele ($x_{ji} = 2$), given that $x_{ji} \neq 0$. Missing values for the SNPs used as predictors are temporarily imputed by randomly sampling a genotype value with probabilities according to the genotype frequencies of that SNP.

Once the two LPMs are fitted, they are used to impute the missing values for *j*. For each individual *i* with missing x_{ji}, the predicted probabilities $\hat{\pi}(x_{ji} = 0)$ and $\hat{\pi}(x_{ji} = 1 \mid x_{ji} \neq 0)$ are computed using LPM1 and LPM2 respectively, from which $\hat{\pi}(x_{ji} = 1)$ and $\hat{\pi}(x_{ji} = 2)$ are then computed. A value for x_{ji} is then randomly selected according to these three predicted marginal probabilities.

A simulation was performed to evaluate the quality of the imputation. From the CD data 50 genes with between 10 and 30 SNPs were selected, more or less evenly spread across the spectrum of gene p-values. At random, a fraction *F* of all the genotypes was set to missing, and then the MAGMA PC regression gene analysis was run with the imputation procedure as described above. This procedure was repeated 100 times for each value of *F*, and the 5th and 95th quantile of the p-values for each gene p-value are shown in Figure S5. As the figure shows the imputation works quite well, with the lower quantiles (and thus the upper quantiles of the -log10 scale shown in the plots, shown in black) still close to the original p-values. The stronger deviation of the upper quantiles (in blue) make it clear that the missingness will tend to make the analysis somewhat more conservative, which is unsurprising given the fact that relative to the full data situation information is lost. However, since the upper quantiles essentially represent a worst case scenario and the missingness in a well-cleaned

data-set is typically very low, often lower than the fractions used in these simulations, this should have little impact in practice.

Gene correlation matrix for gene-set analysis

In order to correct for LD between genes in the gene-set analysis, the correlation matrix R is computed from the joint sampling distribution of the vector *Z* of gene Z-values. Since the correlations between genes are caused by LD between SNPs which quickly drops off as a function of distance, correlations are only computed for pairs of genes within a certain distance from each other (5 megabases) and are otherwise assumed to be zero; correlations between genes on different chromosomes are always set to zero. This helps to limit the effective size of R. In particular, R becomes a block diagonal matrix, and as a result the inverse and the eigenvalue decomposition of R can both be computed from the inverses and eigenvalue decompositions of the blocks on the diagonal.

Since for each gene *g* the genotype data is projected onto its principal components matrix P_g prior to performing the analysis, the SSM for each gene is proportional to the quadratic form $Y^T P_g P_g^T Y$. Under the null hypothesis of no association, the elements of Y follow independent standard normal distributions and the covariance of the SSM's for two genes *i* and *j* is therefore given by $2 \times \text{Trace}(P_i^T P_j P_j^T P_i)$. The standard deviations of each SSM are equal to $\sqrt{2 \times K_i} \times (N-1)$ for *i* and mutatis mutandis for *j*, with K_i and K_j the number of PCs in each gene and *N* the sample size. All three values are straightforward functions of the genotype data and can therefore be computed very quickly, and the covariance divided by the two standard deviations subsequently gives the correlation between the two genes.

To verify the validity of this approach, the full gene correlation matrix was computed for the PC regression on the CD data. Since MAGMA does not compute this by itself, an empirical sampling distribution of the SSM values per gene was computed based on 4,611 permutations of the phenotype. QQ-plots of the correlations are shown in Figure S6, with expected quantiles computed from a simulated sample of 4,611 permutations for 13,172 independent genes. As panel B shows, the correlations between genes more than 5 megabases apart exactly follow the distribution expected if they were not correlated, indicating that these correlations are simply the product of noise. This can also be seen in Figure S7, which visualises the subset of R corresponding to chromosomes 5 and 6. Correlations with an absolute value smaller than 0.05 have been set to 0 to remove noise from the plot, and as the figure shows virtually all the remaining non-zero correlations fall inside the area covered by the 5 megabase maximum range.

SNP-wise gene analysis

Structure of the analysis

In the SNP-wise gene analysis implemented in MAGMA, first the individual SNPs in a gene are analysed to produce a p-value for each SNP. These p-values are then transformed and combined into a gene test-statistic. Depending on the gene test-statistic, either an approximation to the sampling distribution or phenotype permutation is used to compute the gene p-value. The SNP-wise gene analysis can be used as input for the gene-set analysis in the same way as the PC regression model.

The SNP-wise models differ from each other in both the transformation applied to the SNP p-values and the manner in which they are subsequently aggregated to a gene test-statistic. SNP p-values can be transformed to either χ^2 statistics or standard-normal *Z* statistics, with lower p-values corresponding to higher statistics and vice versa. The SNP statistics are then combined into one of several available gene test-statistics described below.

Gene test-statistics

A number of different ways of constructing the gene test-statistic from the SNP statistic (*Z* or χ^2) have been implemented in MAGMA. Either the mean SNP statistic, the top SNP statistic or the (weighted) mean of several of the top SNP statistics can be used. For this last option, the number of SNPs to use can be specified as either a fixed number, or as a proportion of the number of SNPs in the gene.

When using the mean SNP statistic, optional weights can be used to partially correct for the dependency between SNPs. These weights are based on the SNP statistic correlation matrix R, with w_i equal to the mean of the elements of the *i*th column of R^{-1}. When using the permutation approach the matrix R is computed as the correlation matrix of the joint empirical sampling distribution. When using the approximate sampling distribution approach, it is computed directly from the genotype data as described in the next section.

Computation of approximate sampling distribution p-values

For the weighted and unweighted mean Z and mean χ^2 gene test-statistics, an approximation to the sampling distribution can be computed. For both Z and χ^2 this requires a correlation matrix R for the SNP statistic. Analogous to the gene correlation matrix used in the gene-set analysis (see 'Implementation Details' above) the correlations between the gene SSM values of the regression

model are used. For single SNPs, this reduces to the square of the correlation between the SNP genotype values.

For the mean Z gene test-statistics the approximate sampling distribution is based on basic properties of the multivariate normal distribution. The sum of a set random variables with a joint multivariate normal distributions itself has a univariate normal distribution with known mean and variance. Here, under the null hypothesis of no association the individual SNP Z-values z_i have a standard normal distribution, and thus their joint distribution can be assumed to be a multivariate normal distribution with a mean of 0 and covariance equal to the correlation matrix R. The gene test-statistic is therefore equal to $T = \sum_i^K w_i z_i$, with w_i the weight for SNP *i*, and there the sampling distribution of T is a normal distribution with a mean of 0 and variance $V = \sum_{i=1}^K w_i^2 + 2\sum_{i=1}^K \sum_{j<i} w_i w_j R_{ij}$.

The procedure used for the mean χ^2 gene test-statistics is based on the approach described by Brown (1975)[20,21], which approximates the sampling distribution of a weighted sum of dependent χ^2 random variables by that of a scaled χ^2 variable. Under the null hypothesis of no association the SNP p-values have a uniform sampling distribution, and therefore $-2\log(p_i) \sim \chi^2_{(2)}$. For the test-statistic $T = -2\sum_i^K w_i \log(p_i)$ it is then assumed that $T \sim c\chi^2_{(f)}$, and the constants *c* and *f* are then determined by equating the first two moments of T and this scaled χ^2 distribution. As such $c = \frac{\mathrm{Var}(T)}{2\,\mathrm{E}(T)}$ and $f = \frac{2\,\mathrm{E}(T)^2}{\mathrm{Var}(T)}$, with $\mathrm{E}(T) = \sum_{i=1}^K w_i$, $\mathrm{Var}(T) = 4\sum_{i=1}^K w_i^2 + 2\sum_{i=1}^K \sum_{j<i} w_i w_j C_{ij}$ and $C_{ij} = \mathrm{Cov}(-2\log(p_i), -2\log(p_j))$. These covariance values can in turn be approximated with $C_{ij} = R_{ij}(3.25 + 0.75R_{ij})$.

Computation of permutation p-values

For all gene test-statistics an empirical p-value can be computed based on permutation of the phenotype. If a top-SNP statistic is used no approximate sampling distribution is available and permutation is the only option. When computing permutation p-values, by default an adaptive procedure is used to determine the number of permutations to use. For each gene MAGMA generates 1,000 permutations, then checks the number P_+ of permutations with a gene test-statistic greater than the observed gene test-statistic. If this number is greater than the prespecified threshold P_{thresh} (by default, $P_{\mathrm{thresh}} = 10$) the empirical p-value is computed and the analysis moves

to the next gene. Otherwise, the number of permutations is increased to 5,000 and P_+ is checked again. The process continues (checking at 10,000, 50,000, 100,000, and so on) until either $P_+ > P_{\text{thresh}}$ or the maximum number of permutations is reached (by default, the maximum is 1,000,000).

Analysis of summary statistics

The SNP-wise gene analysis can be used with both raw genotype data as well as with summary SNP p-values obtained from a previous single-marker analysis. In the latter case, an additional reference data-set must be provided to compute the empirical sampling distribution of the gene test-statistic. This is needed to account for the LD between SNPs. More information about the choice of reference data can be found below in the section 'Reference data for p-value only analysis'.

The gene analysis of summary statistics proceeds in largely the same way as analysis on raw genotype data. The observed gene test-statistic is computed from the provided SNP p-values, using only SNPs present in the reference data as well. The reference data-set takes the place of the raw genotype data, and if permutation is a simulated phenotype is drawn from the standard normal distribution. This simulated phenotype is then permuted to generate the empirical sampling distribution and compute the gene p-value. If an approximate sampling distribution is used, the reference data is only used to estimate the SNP statistic correlation matrix.

Reference data for p-value only analysis

Since the gene analysis on summary statistics requires a reference data-set to account for the LD between SNPs, a number of analyses on the CD SNP p-values (computed using PLINK) were run with different reference data-sets using the unweighted mean χ^2 gene test-statistic. These were then compared to the raw data SNP-wise analysis with the same gene test-statistic (Figure S8).

The first plot shows the analysis using the CD data as reference, which as would be expected yields highly similar results. The small differences with the raw data analysis can be attributed to a combination of permutation noise and slight differences in computing the SNP p-values. For the most part the 1,000 Genomes European does just as well as reference data as the CD data itself. The only difference is that since not all SNPs from the CD data are present in the 1,000 Genomes data, 97 genes cannot be analysed.

The HapMap European data-set does not perform as well as the 1,000 Genomes data, with more pronounced differences in gene p-values and 375 genes missing due to its less extensive coverage. There is no indication of a bias in the p-values however, suggesting that it could still serve as a reasonable reference data-set. The same cannot be said for the African HapMap sample

however, which has a significant downward bias in the gene p-values, as well as even more missing genes (623). This bias is likely caused by the overall lower LD in African populations, which results in an underestimation of the variance of the sampling distributions.

Extensions to the model

Gene by environment interaction

MAGMA offers the possibility of adding a gene-environment (or more generally, gene-covariate) interaction component to the gene analysis. To do so, first all SNPs in the genotype data matrix X_g for a gene *g* are centered and the interactor covariate *C* is standardized. For each SNP *j* the element-wise product of X_{gj} and *C* (i.e., the vector with elements $X_{gji} \times C_i$ for each individual *i*) is computed and added to X_g. In other words, all the SNP by covariate interactions are added to the model. The PC regression then proceeds as normal (adding *C* as a covariate), computing the PCs from the augmented X_g, and performing the F-test on the vector of its coefficients. This thus results in a joint test of both the genetic main effect and the gene-environment interaction component. At present, only one covariate can be used as an interactor at a time.

Rare variants

When analysing data containing rare variants, rare variants within a gene can be aggregated to improve statistical power. To do so, for each gene *g* a new variable $R_g = \sum_r X_r$ is constructed. Here, *r* indexes over the rare variants in the gene, with 'rare' defined according to a user-specified MAF threshold. Since minor allele coding is used, R_{gi} for an individual *i* reflects the number of minor alleles on rare variants that individual has for gene *g*.

In the analysis, the new R_g variable is used instead of the rare variants themselves. The advantages of this approach is that this uses only a single parameter in the model rather than a parameter for each rare variant. Under the assumption that the minor alleles for these variants have the same direction of effect, this will improve the power to detect associations.

When a gene contains many rare variants aggregating all those variants to a single compound variable may be too severe, because the signal of associated variants may be drowned out by the noise of too many non-associated variants. To counteract this, a maximum *m* can be set to the number of rare variants that are aggregated into R_g. If a gene contains more than *m* rare variants, multiple aggregate variables $R_g^{(j)}$ are constructed, each corresponding to no more than *m* variants.

Gene and gene-set meta-analysis

To facilitate the analysis of multiple independent data-sets, MAGMA also has an option for fixed effects meta-analysis for both the gene and the gene-set analysis. For both, Stouffer's weighted Z-score method is used[27], with weight w_d for each data-set *d* set to the square root of its sample size. Let p_d be the gene or gene-set p-value for data-set *d*, and define $z_d = \Phi^{-1}(1 - p_d)$ with Φ^{-1} the probit function. These Z-values can now be combined across data-sets to obtain $z_C = \sum_d w_d z_d / W_C$, where $W_C = \sum_d w_d^2$. Under the assumption that the data-sets are independent, z_C has a standard normal distribution under the null hypothesis of no association for that gene or gene-set in any of the data-sets, and therefore the meta-analytic p-value can be computed as $p_C = 1 - \Phi(z_C)$, where Φ is the cumulative normal distribution function.

Additional references

[27] Whitlock MC (2005) Combining probability from independent tests: the weighted Z-method is superior to Fisher's approach. J Evolution Biol 18: 1368-1373.
