# Supplementary material for: MAGMA: Generalized Gene-Set Analysis of GWAS Data
Source: PLoS Comput Biol. 2015 Apr 17;11(4):e1004219. doi: 10.1371/journal.pcbi.1004219 (PMC4401657; doi:10.1371/journal.pcbi.1004219)
Supplement: S2 File — (PDF) [file pcbi.1004219.s002.pdf]

Supplemental figures and tables

MAGMA: Generalized gene-set analysis of GWAS data

Table S1. Mean type 1 error rates for gene analysis

Table S2. Mean type 1 error rates for gene-set analysis

Table S3. Mean power for different gene analysis models in post hoc power analysis

Table S4. Power for individual genes in the second post hoc power analysis

Table S1: Mean type 1 error rates (with SD) for gene analysis.

Model	Mean type 1 error rate (SD)
MAGMA-main	0.049 (.007)
MAGMA-mean	0.049 (.007)
MAGMA-top	0.050 (.007)
MAGMA-pval	0.048 (.008)
MAGMA-unwZ	0.049 (.007)
MAGMA-wtdZ	0.049 (.008)

Based on 1,000 permutations of the phenotype. Error rates were computed individually for each gene in the CD data, then averaged.

Table S2: Mean type 1 error rates (with SD) for gene-set analysis.

	Global null model		Polygenic null model	
Gene-sets	Self-contained	Competitive	Self-contained	Competitive
MSigDB	0.051 (.007)	0.052 (.007)	0.088 (.028)	0.051 (.009)
Size = 10, clump = 1	0.050 (.007)	0.051 (.008)	0.073 (.011)	0.051 (.009)
Size = 10, clump = 2	0.049 (.007)	0.050 (.008)	0.071 (.011)	0.050 (.009)
Size = 10, clump = 5	0.049 (.007)	0.049 (.008)	0.070 (.013)	0.051 (.010)
Size = 100, clump = 1	0.051 (.007)	0.053 (.007)	0.131 (.015)	0.052 (.008)
Size = 100, clump = 2	0.050 (.007)	0.051 (.007)	0.124 (.014)	0.051 (.008)
Size = 100, clump = 5	0.049 (.007)	0.049 (.007)	0.116 (.016)	0.050 (.009)

For MSigDB gene-sets as well as simulated gene-sets. Gene-sets were simulated by randomly sampling a specified number of genes from the CD data, either individually or in sets of 2 or 5 adjacent genes. Type 1 error rates were evaluated under both a global null model (no associated SNPs in the data) and a polygenic null model (1,000 associated SNPs randomly scattered across the genome, together explaining 50% of phenotypic variance). The rates shown are the mean error rates averaged over all the gene-sets, based on 1,000 simulated phenotypes.

Table S3: Mean power for different gene analysis models in post hoc power simulations.

	Simulation 1				Simulation 2	
	R^2 = 0.1%		R^2 = 0.5%		R^2 = 0.1%	R^2 = 0.5%
Method	Low LD	High LD	Low LD	High LD		
MAGMA-main	0.32 (.03)	0.32 (.02)	0.97 (.01)	0.97 (.01)	0.35 (.03)	0.98 (.00)
MAGMA-mean	0.19 (.04)	0.41 (.08)	0.90 (.04)	0.98 (.02)	0.19 (.12)	0.62 (.42)
MAGMA-top	0.38 (.03)	0.41 (.02)	0.99 (.00)	0.99 (.00)	0.20 (.11)	0.63 (.39)

Values shown are mean (SD) power of 10 genes. Simulation 1 compares power to detect effects in isolated SNPs (low LD) and effects in SNPs in LD with 4 other SNPs (high LD). Simulation 2 shows power to detect obscured two-marker effect.

Table S4: Power for individual genes in the second post hoc power analysis.

	**Correlation**		**R^2 = 0.1%**			**R^2 = 0.5%**	
		**MAGMA-main**	**MAGMA-mean**	**MAGMA-top**	**MAGMA-main**	**MAGMA-mean**	**MAGMA-top**
Mean	-	0.348	0.190	0.205	0.980	0.619	0.634
SD	-	0.027	0.118	0.113	0.005	0.420	0.385
Gene 1	0.17	0.33	0.28	0.27	0.98	0.96	0.95
Gene 2	0.99	0.36	0.05	0.06	0.98	0.05	0.07
Gene 3	0.21	0.41	0.33	0.36	0.99	0.95	0.96
Gene 4	0.56	0.36	0.16	0.21	0.98	0.83	0.79
Gene 5	0.88	0.35	0.06	0.09	0.98	0.13	0.23
Gene 6	0.12	0.34	0.29	0.31	0.98	0.97	0.96
Gene 7	0.99	0.32	0.05	0.06	0.98	0.05	0.08
Gene 8	0.36	0.32	0.27	0.28	0.98	0.96	0.93
Gene 9	0.52	0.35	0.32	0.27	0.98	0.96	0.93
Gene 10	0.77	0.36	0.10	0.13	0.99	0.33	0.45

The ‘correlation’ column gives the correlation between the two SNPs in the gene on which the multi-marker effect was based.
