# Supplementary figures and images for: MAGMA: Generalized Gene-Set Analysis of GWAS Data
Source: PLoS Comput Biol. 2015 Apr 17;11(4):e1004219. doi: 10.1371/journal.pcbi.1004219 (PMC4401657; doi:10.1371/journal.pcbi.1004219)

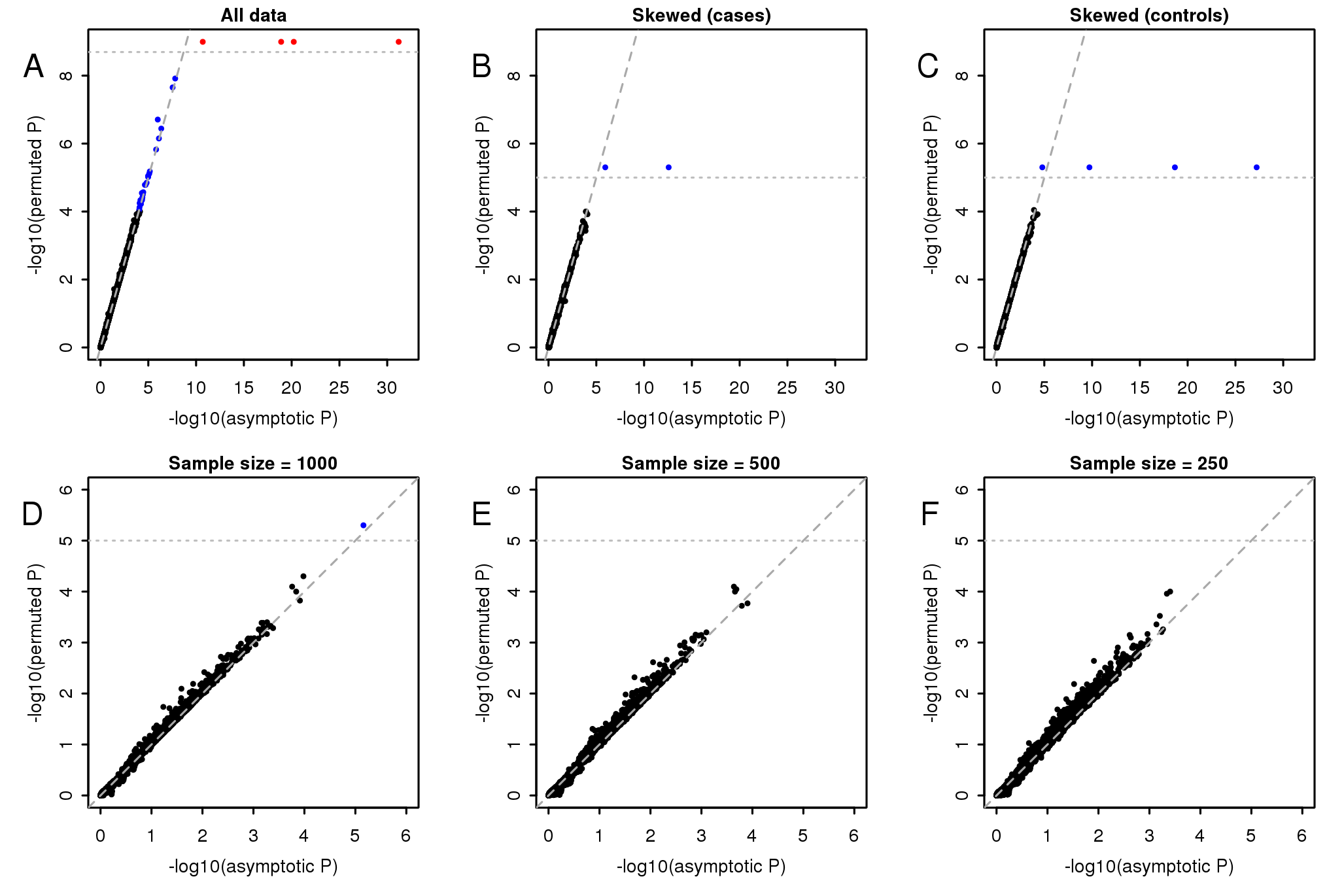

Supplement: S1 Fig — Empirical p-values were obtained for the CD data PC regression gene analysis by permutation of the F-statistic (A), in order to verify the accuracy of the asymptotic F-test p-values. An initial 100,000 permutations were computed for each gene. For genes with a very low initial empirical p-value (shown in blue and red) the number of permutations was increased to about 500 million to refine the empirical p-value. The dashed horizontal line indicates the lowest possible non-zero permutation p-value, genes with an empirical p-value of 0 are shown at half that minimum p-value in the plot (in red). The process was repeated using a subsample of the CD data skewed 4:1 towards cases (B) or controls (C); and with evenly divided subsamples of N = 1000 (D), N = 500 and N = 250. Only the initial 100,000 permutations were performed for these analyses, genes with an empirical p-value of 0 are again shown at half the minimum non-zero p-value (in blue). (TIFF) [file pcbi.1004219.s004.tiff]

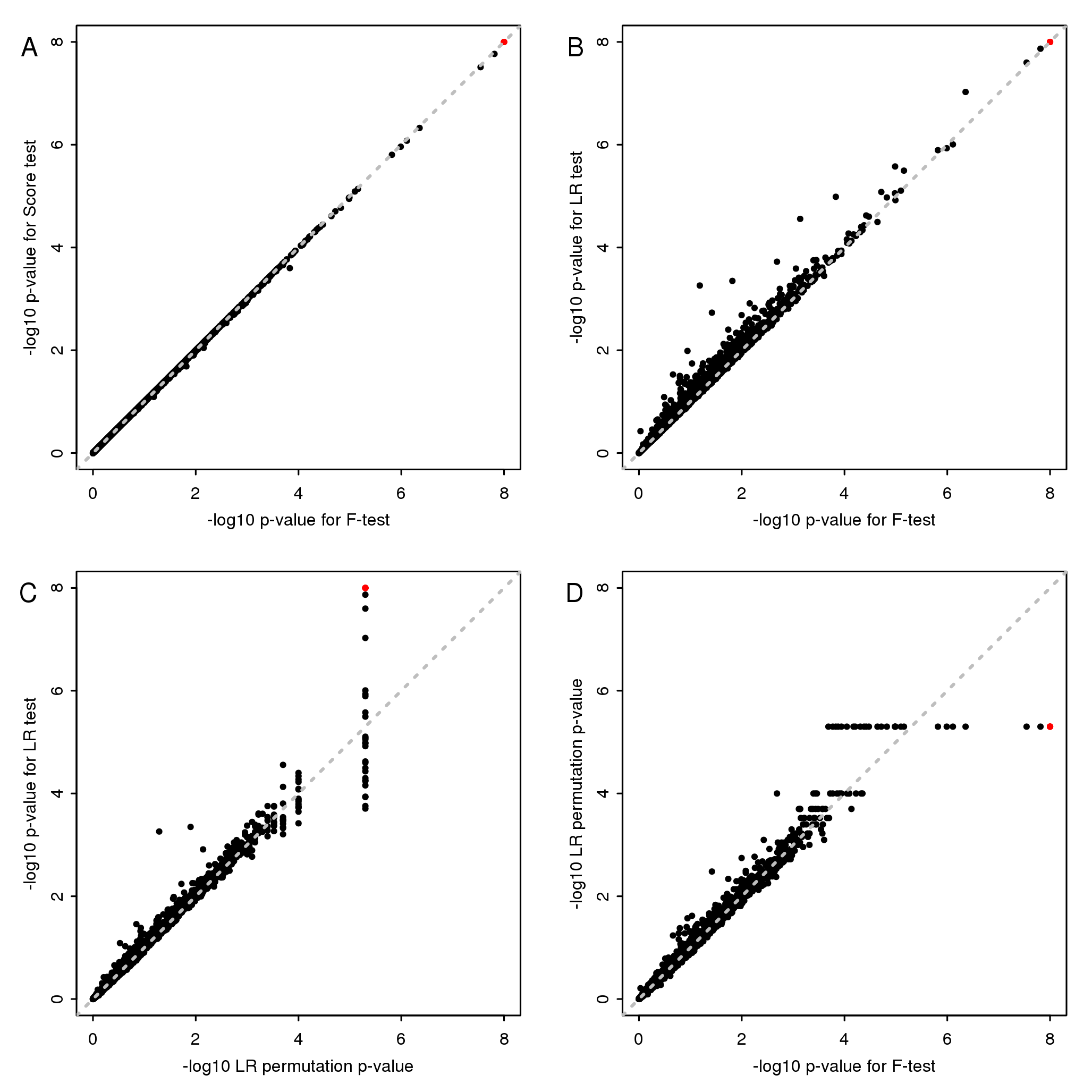

Supplement: S2 Fig — Gene p-values were computed using a logistic regression model to compare against the linear regression model used in MAGMA. P-values were computed using either a Score test (A) or a Likelihood Ratio test (B). Because the Likelihood Ratio test appeared to have significantly more power than both the Score test and the MAGMA F-test, empirical p-values for the Likelihood Ratio test were computed by generating up to 10,000 permutations of the Likelihood Ratio statistic. This was compared to the asymptotic Likelihood Ratio test p-values (C), revealing a downward bias in the asymptotic p-values. The empirical p-values were then compared to the MAGMA F-test p-values (D), which shows that the apparent power advantage of the Likelihood Ratio test in (B) was due to the bias in the p-values. (TIFF) [file pcbi.1004219.s005.tiff]

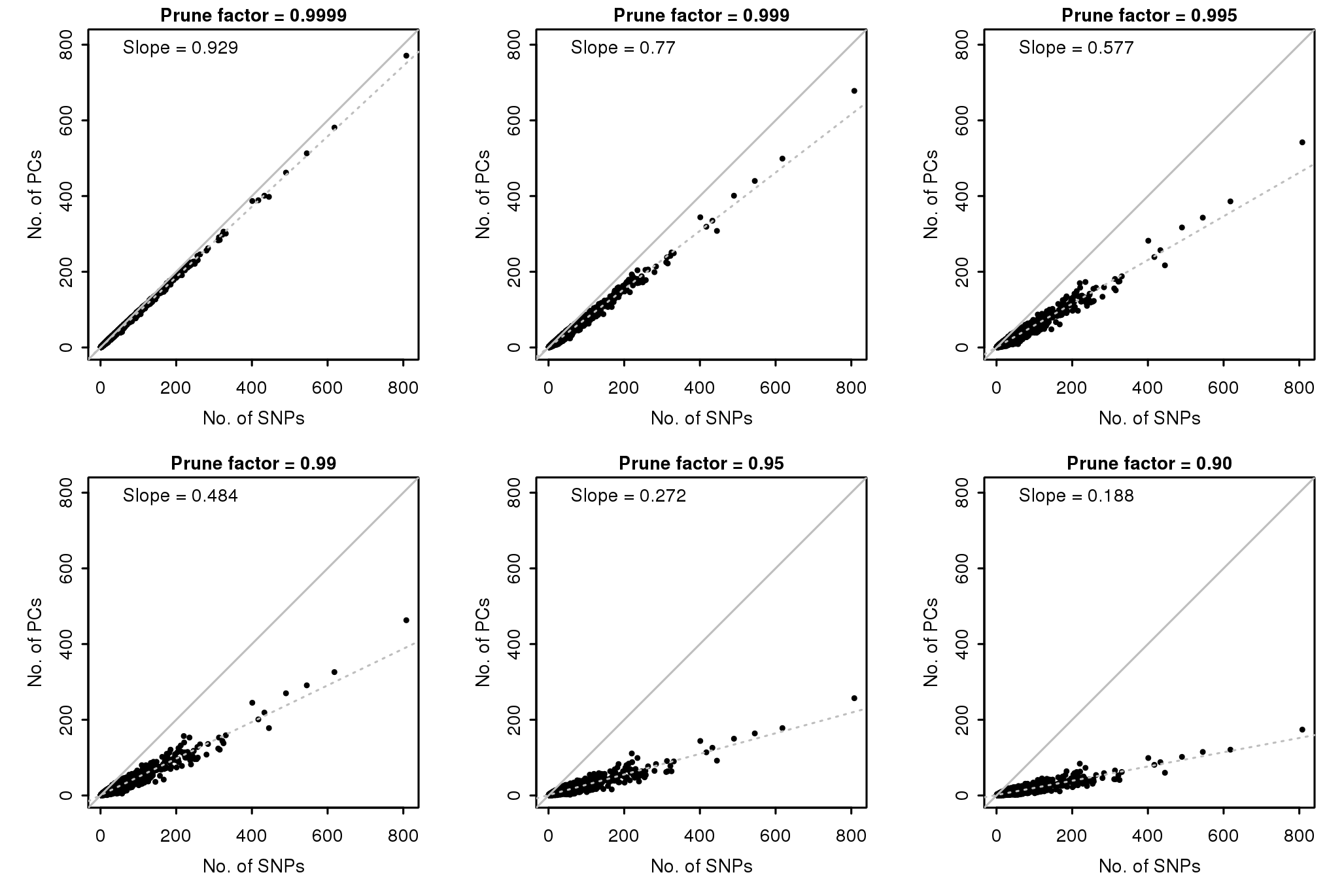

Supplement: S3 Fig — The pruning implemented in MAGMA was applied to the genes in the CD data at different levels of the prune factor f (default is 0.999), which reflects the proportion of the total variance in the raw genotype data that is retained after pruning. The original number of genotyped SNPs in each gene is plotted against the number of PCs retained after pruning. The regression slope gives an estimate of the average proportion of PCs to SNPs. (TIFF) [file pcbi.1004219.s006.tiff]

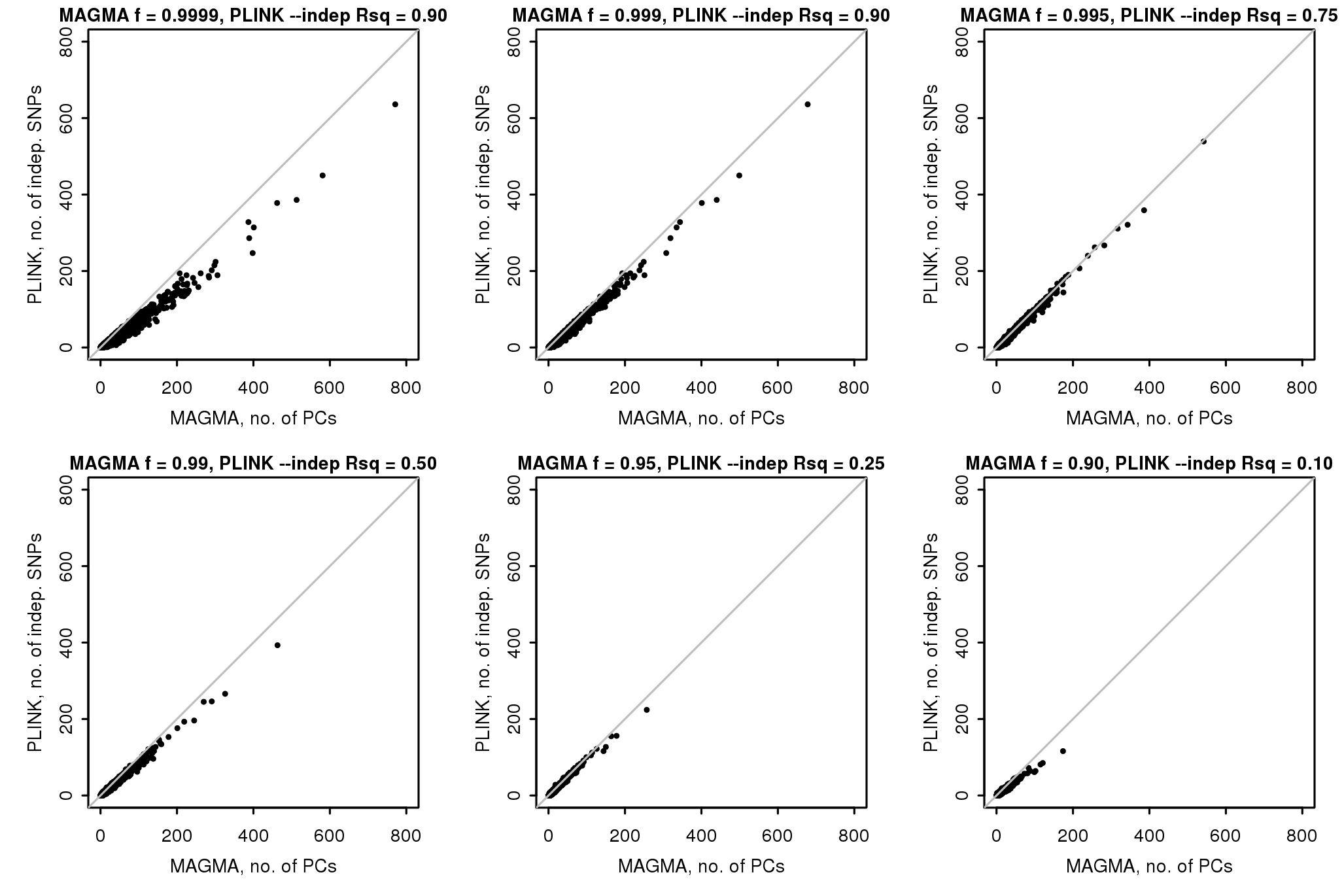

Supplement: S4 Fig — The PLINK—indep option was used to obtain an estimate of the number of independent SNPs at different R 2 values. The number of PCs retained by MAGMA at different values of the pruning factor f is plotted against the number of independent SNPs at the R 2 value that provided the closest match. (TIFF) [file pcbi.1004219.s007.tiff]

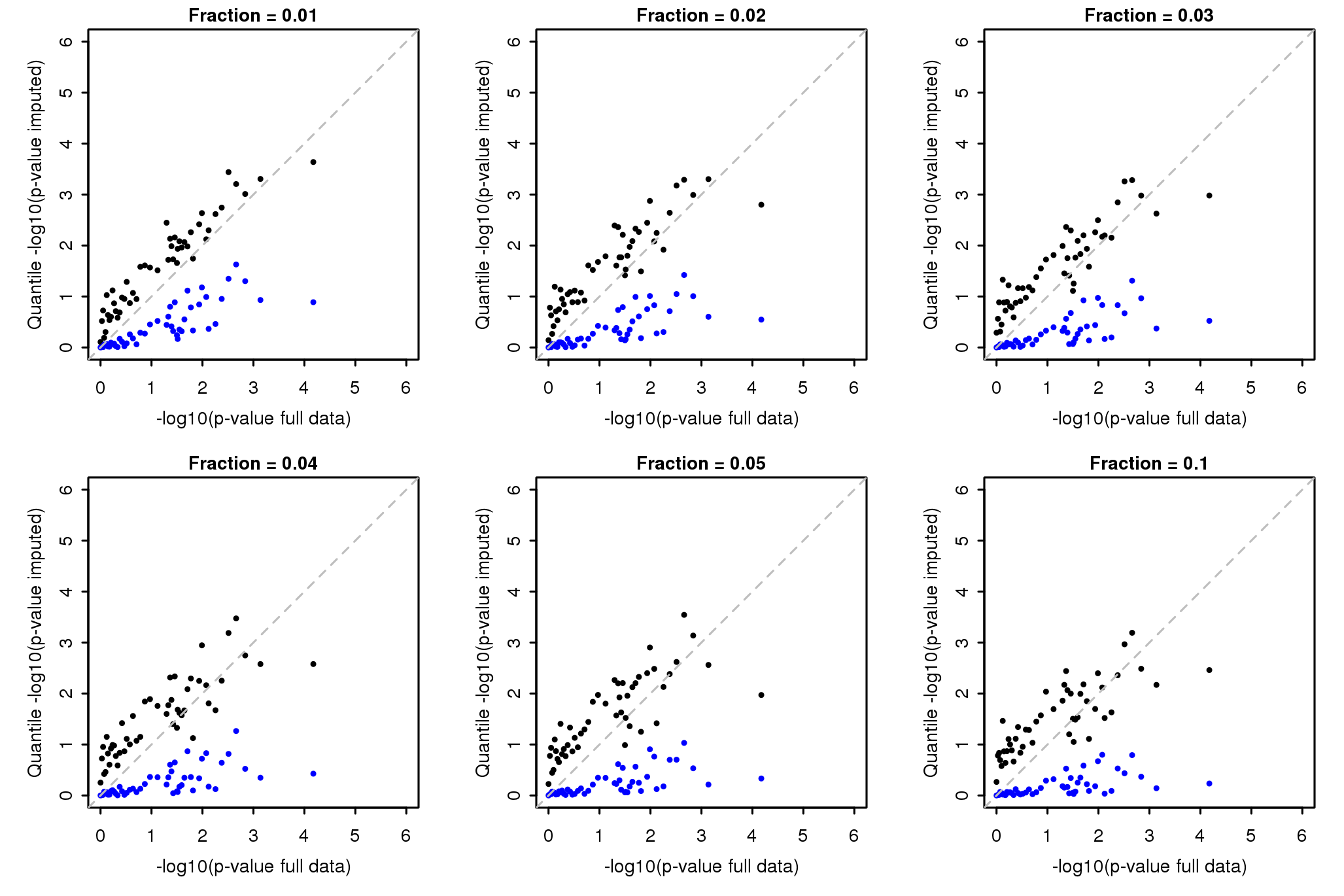

Supplement: S5 Fig — MAGMA needs to impute missing genotype values in order to run the multiple regression model, which is done by single imputation using flanking SNPs. To validate this procedure a subset of genes was selected from the CD data, and genotype values in those genes were set to be missing for a specified fraction of all the genotype values (up to 10%), and gene p-values were then computed after using the imputation to fill in those missing values. Gene p-values were also computed for the original full data. For each fraction, missing data was simulated 100 times for each gene, and the 5th (black) and 95th (blue) quantiles of the p-values of each gene were computed and plotted against that gene’s full data p-value. (TIFF) [file pcbi.1004219.s008.tiff]

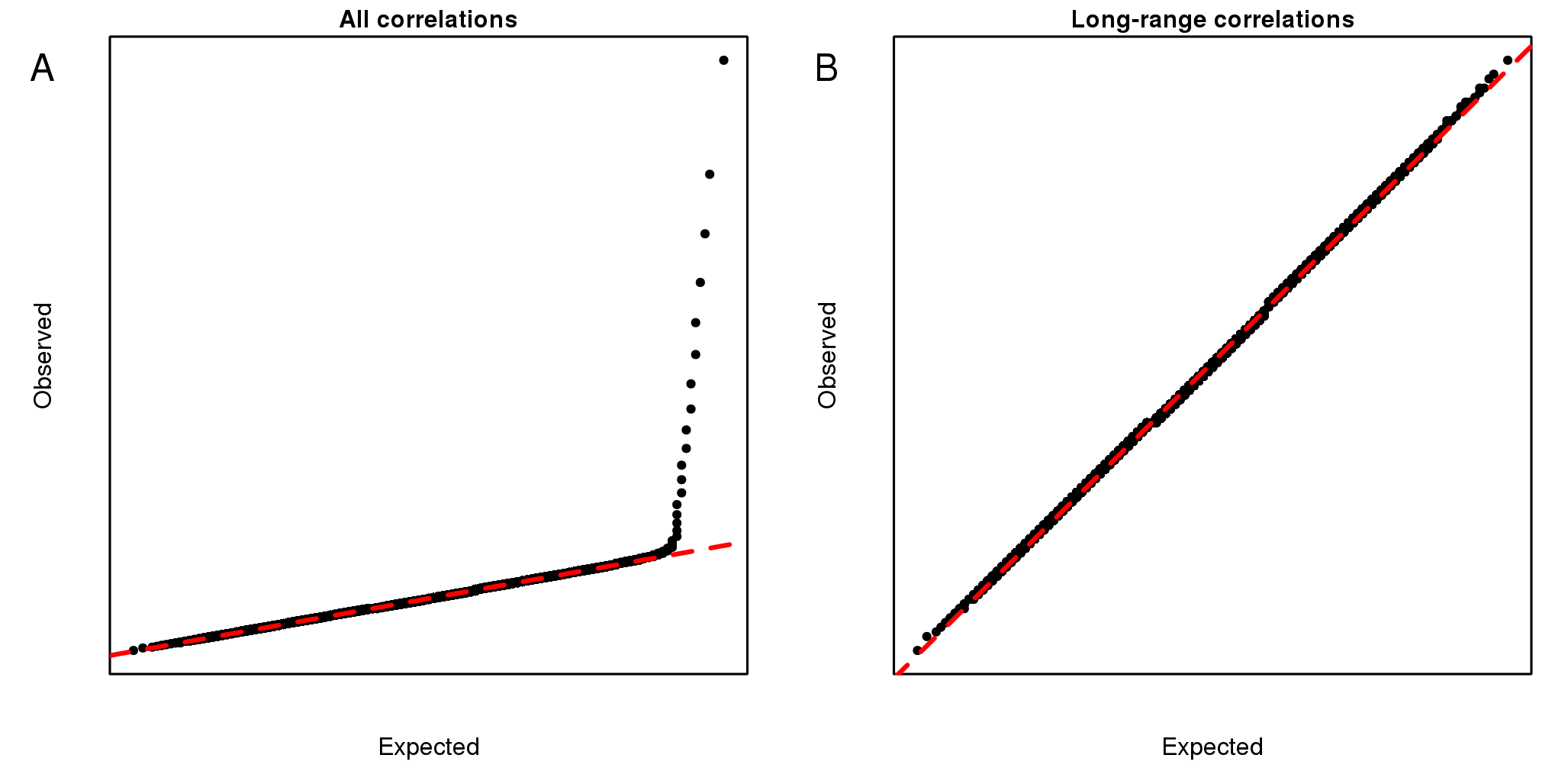

Supplement: S6 Fig — Gene analysis was performed on the CD data, and a joint empirical distribution gene SSM values was generated using 4,611 permutations of the phenotype (since the sample size of the CD data is 4,611). The correlation matrix was then computed from this distribution. In addition, a correlation matrix for 13,172 uncorrelated genes was simulated by generating 4,611 permations for 13,172 genes and computing the correlation matrix. This provides the distribution of correlation coefficients that would be expected if the genes were uncorrelated. A QQ-plot of these expected correlation coefficients are plotted against the observed correlation coefficients in (A), showing a clear surplus of high positive correlations for the CD data genes. A QQ-plot using only correlations between genes more than 5 megabases apart (B) reveals that this is due to short-range correlations only. (TIFF) [file pcbi.1004219.s009.tiff]

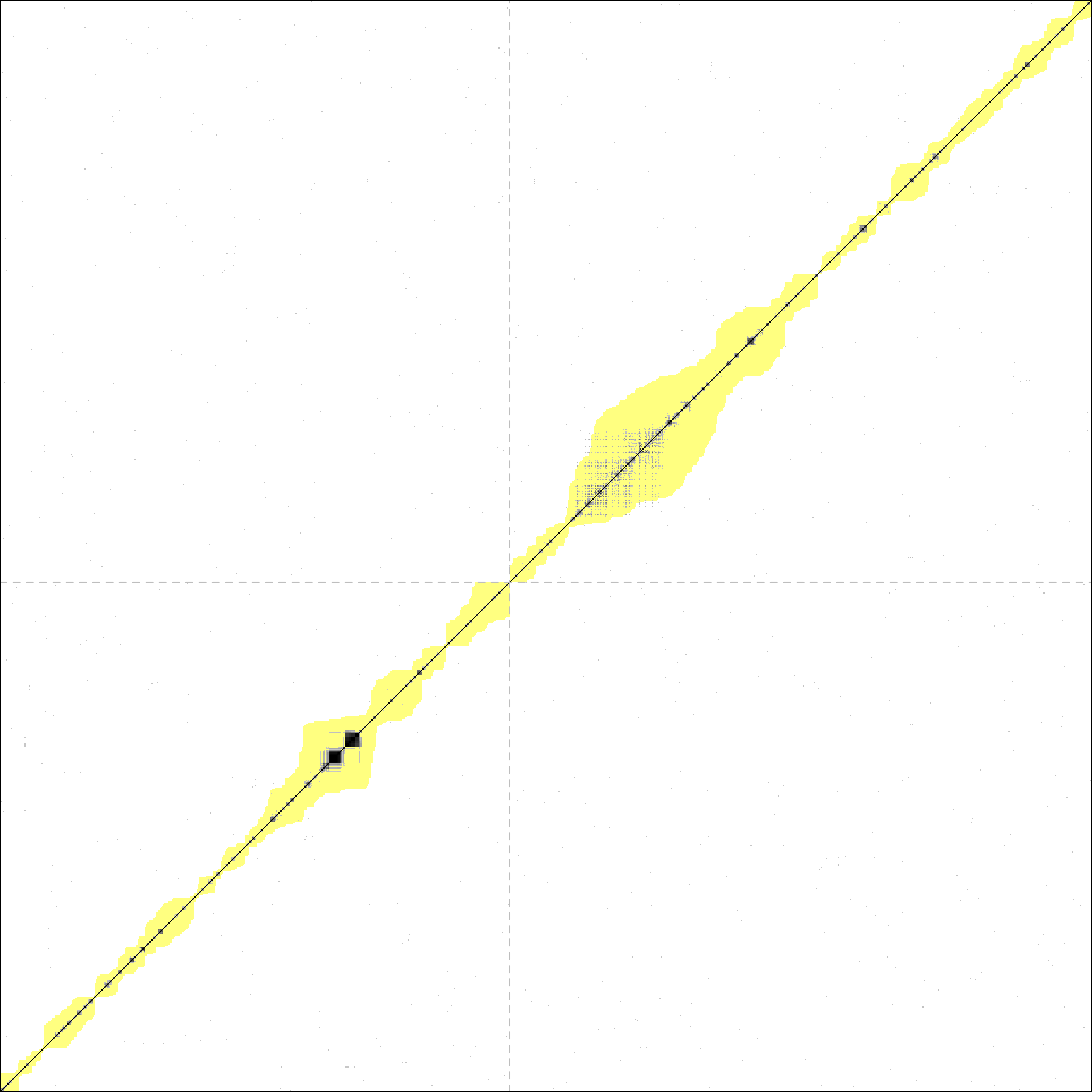

Supplement: S7 Fig — Gene analysis was performed on the CD data, and a joint empirical distribution of the gene SSM values was generated using 4,611 permutations of the phenotype (since the sample size of the CD data is 4,611). The correlation matrix for chromosomes 5 and 6 was plotted, with individual pixels corresponding to a pair of genes and the color (from white to black) proportional to the absolute value of the correlation between those genes. Correlations with absolute value smaller than 0.05 are set to 0 to reduce noise. The yellow area corresponds to genes within 5 megabases of each other, corresponding to gene pairs for which MAGMA computes the correlations (correlations between more distant genes are assumed to be 0); the dashed lines indicate the boundary between the two chromosomes. (TIFF) [file pcbi.1004219.s010.tiff]

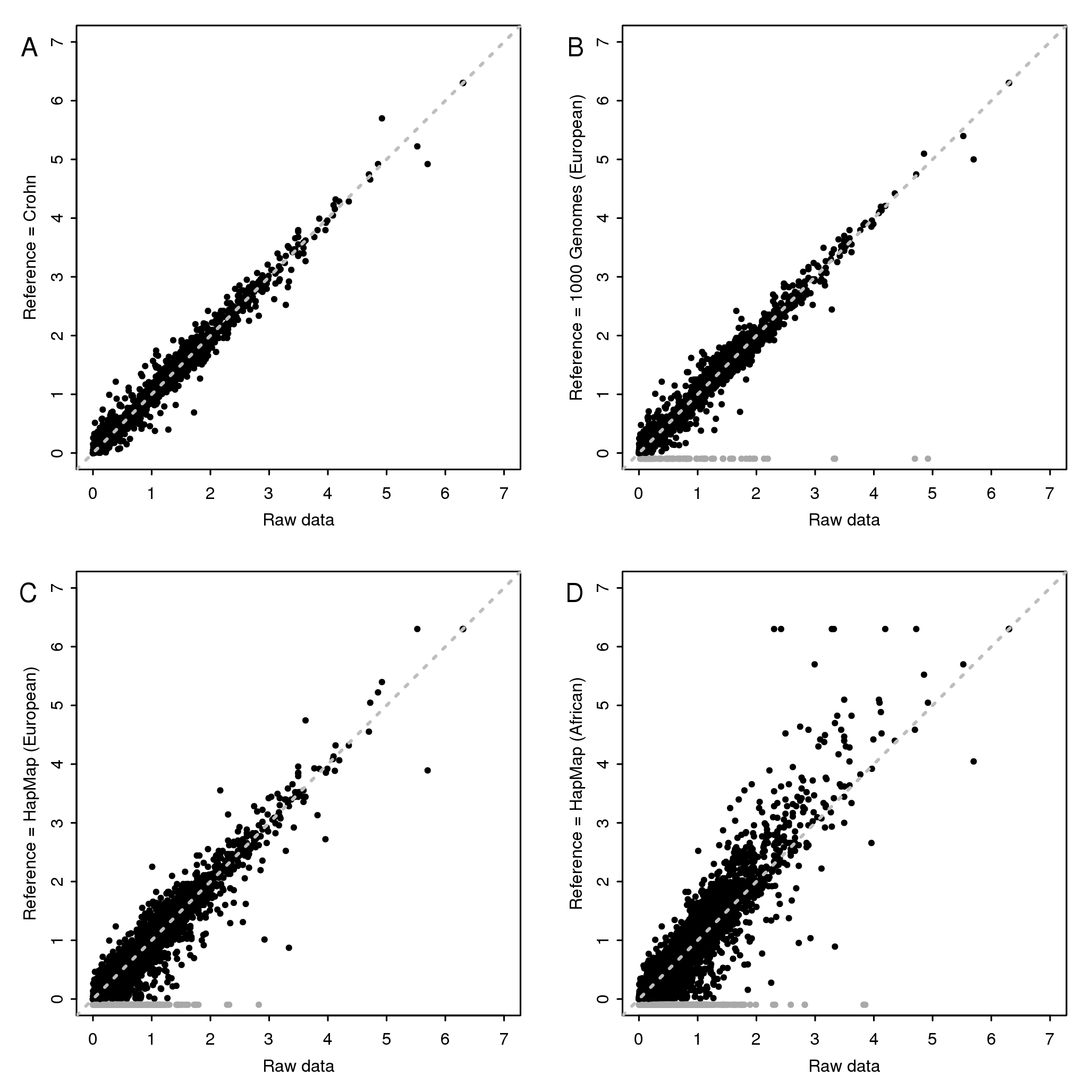

Supplement: S8 Fig — Summary statistics gene analysis of CD data SNP p-values was performed using different reference data-sets, using the SNP-wise mean χ 2 model. This was compared to the same SNP-wise analysis performed on the raw CD genotype data. Grey points correspond to genes not covered by the reference data-set. The reference data-sets used are (A) the CD data itself, (B) the 1,000 Genomes European panel (97 missing genes), (C) the HapMap 3 European panel (375 missing genes) and (D) the HapMap 3 African panel (623 missing genes). (TIFF) [file pcbi.1004219.s011.tiff]

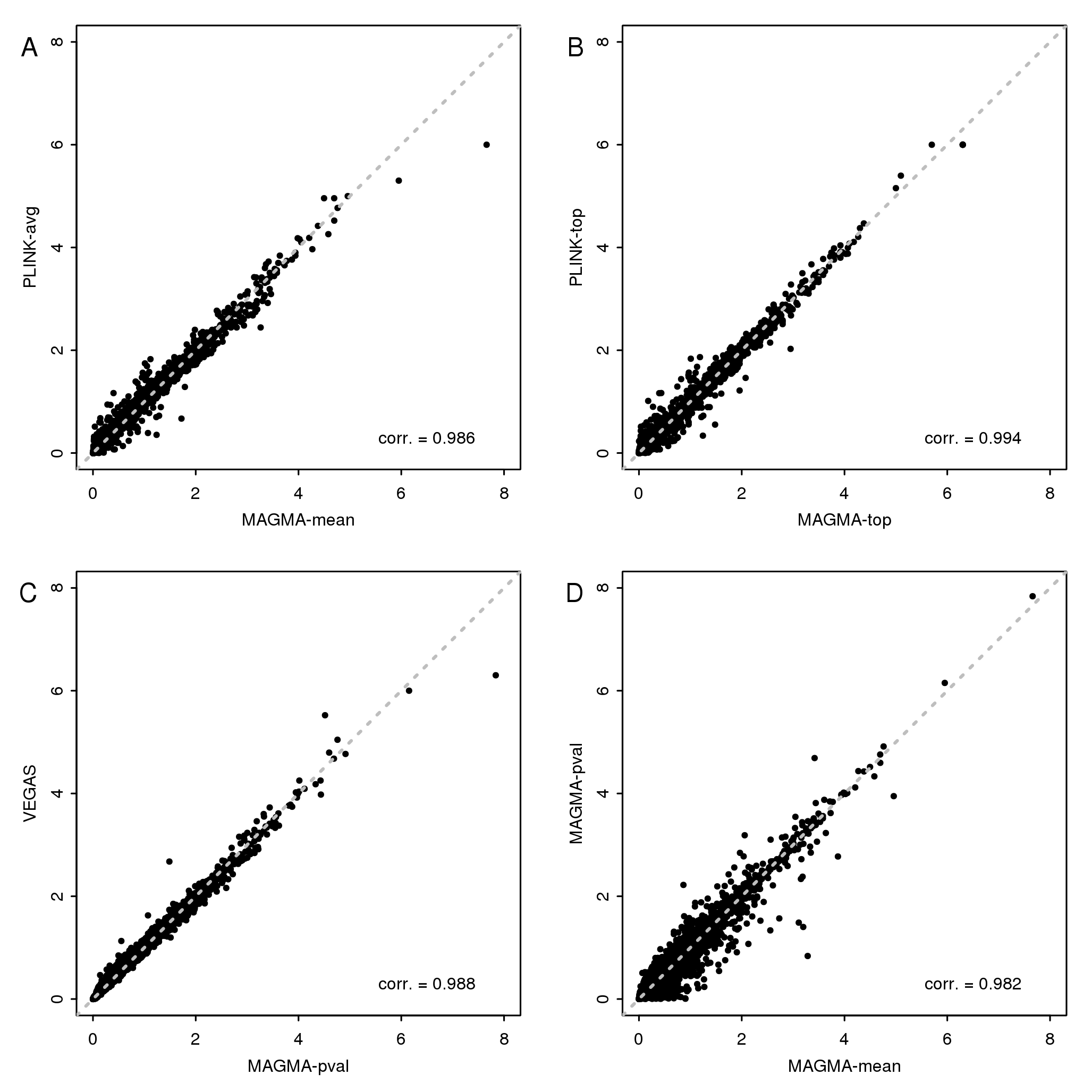

Supplement: S9 Fig — Gene -log10 p-values from the CD data gene analysis for equivalent gene test-statistics implemented in different tools. The gene test-statistics used are (A) the mean χ 2 statistic in MAGMA and PLINK, (B) the top χ 2 statistic in MAGMA and PLINK, (C) the mean χ 2 statistic in MAGMA and VEGAS with analysis based on SNP p-values and HapMap 3 reference data and (D) the mean χ 2 statistic in MAGMA on raw data and with analysis based on SNP p-values and HapMap 3 reference data. (TIFF) [file pcbi.1004219.s012.tiff]

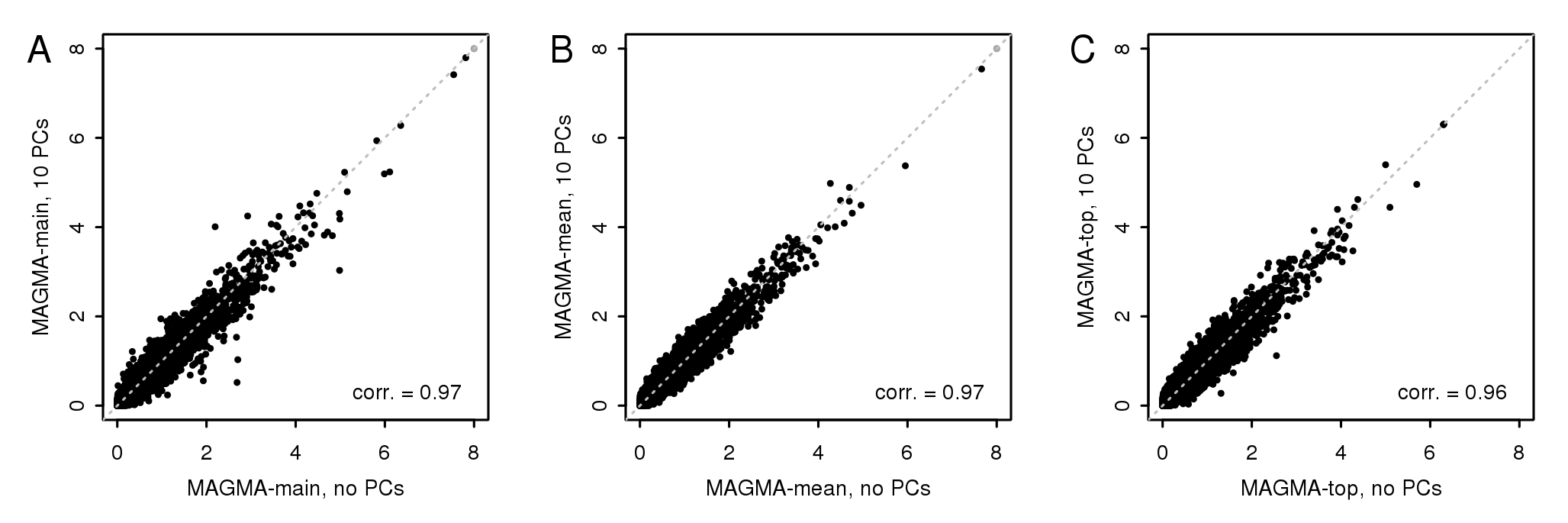

Supplement: S10 Fig — Gene -log10 p-values from the CD data gene analysis for the three MAGMA gene analysis models with 10 PCs as covariates to correct for stratification, and without. P-values below 10–8 are truncated to 10–8 (grey points) to preserve the visibility of the other points. (TIFF) [file pcbi.1004219.s013.tiff]

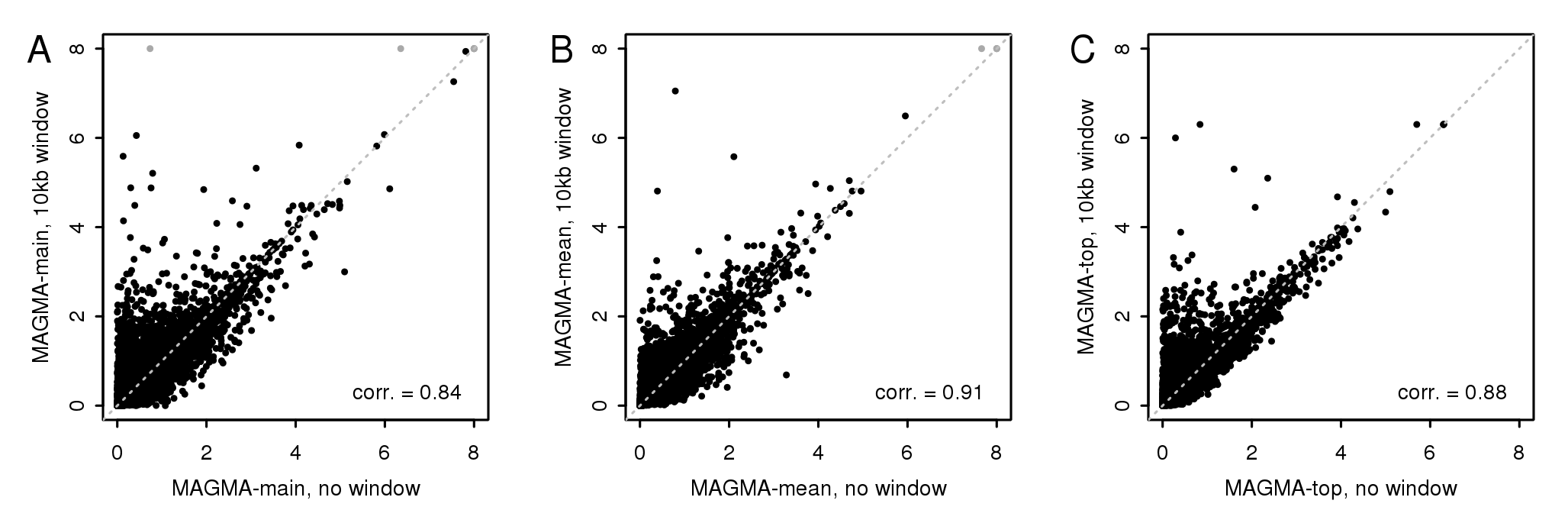

Supplement: S11 Fig — Gene -log10 p-values from the CD data gene analysis for the three MAGMA gene analysis models with additional 10 kilobase window around the transcription start and stop sites, and without. Genes only present in the 10 kilobase window analyses are omitted. P-values below 10–8 are truncated to 10–8 (grey points) to preserve the visibility of the other points. (TIFF) [file pcbi.1004219.s014.tiff]

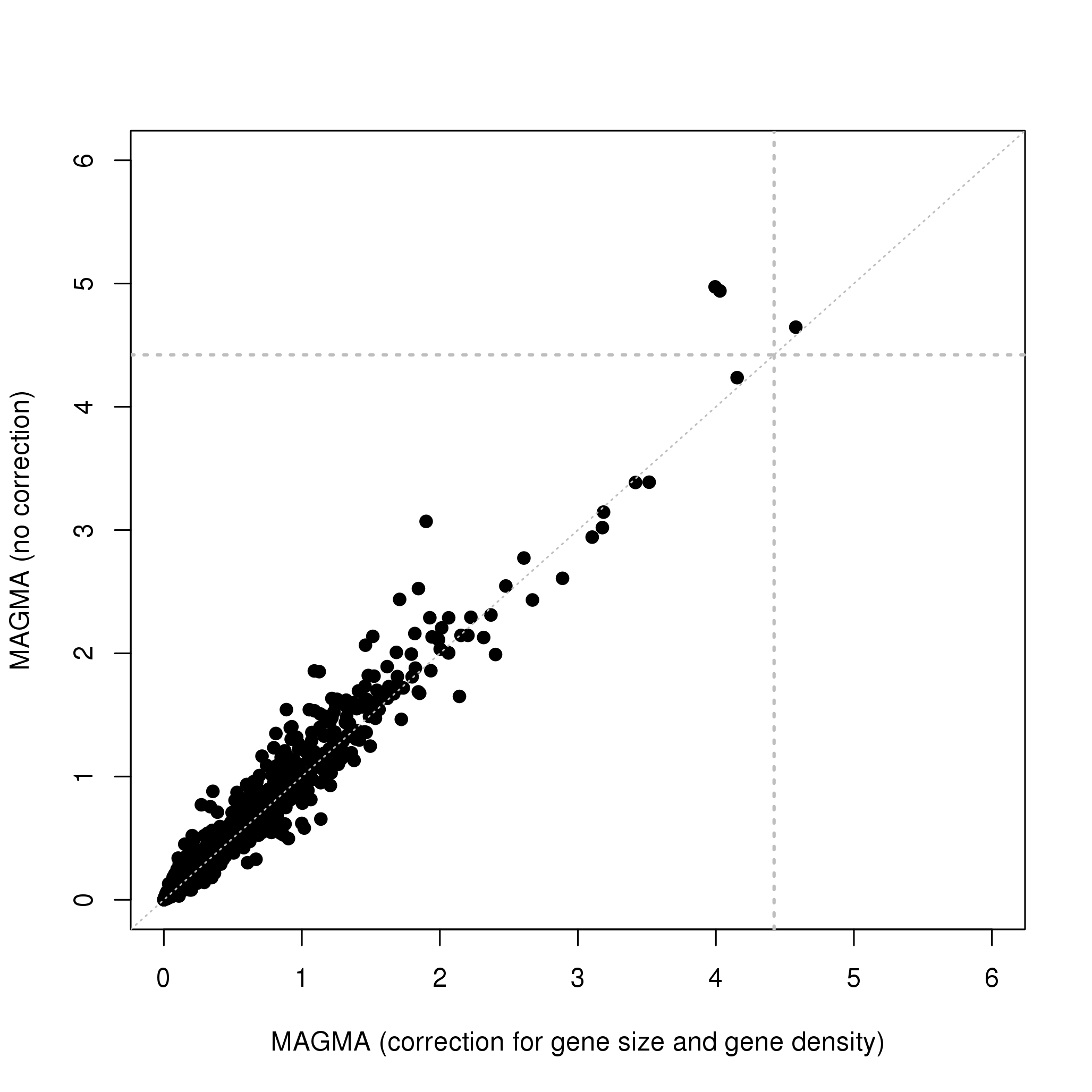

Supplement: S12 Fig — Gene -log10 p-values from the CD data analyses. When the correction is turned on (the default setting), the gene-set effect is conditioned on gene size and gene density. Grey dashed lines represent the Bonferroni-corrected significance threshold. The effective size of the gene (number of PCs in the gene after pruning) is used as a measure of gene size, the ratio of effective size and total number of SNPs as a measure of gene density. The correction is achieved by entering gene size and gene density, as well as the log of both, as predictors in the generalized gene-set analysis model alongside the gene-set indicator variable. (TIFF) [file pcbi.1004219.s015.tiff]
